# Complete Genome Sequence Analysis of *Enterobacter* sp. SA187, a Plant Multi-Stress Tolerance Promoting Endophytic Bacterium

**DOI:** 10.3389/fmicb.2017.02023

**Published:** 2017-10-20

**Authors:** Cristina Andrés-Barrao, Feras F. Lafi, Intikhab Alam, Axel de Zélicourt, Abdul A. Eida, Ameerah Bokhari, Hanin Alzubaidy, Vladimir B. Bajic, Heribert Hirt, Maged M. Saad

**Affiliations:** ^1^Biological and Environmental Sciences and Engineering Division, King Abdullah University of Science and Technology, Thuwal, Saudi Arabia; ^2^Computational Bioscience Research Center, King Abdullah University of Science and Technology, Thuwal, Saudi Arabia

**Keywords:** *Indigofera argentea*, root endophytes, plant–microbe interaction, plant growth-promoting bacteria (PGPB), salt stress

## Abstract

*Enterobacter* sp. SA187 is an endophytic bacterium that has been isolated from root nodules of the indigenous desert plant *Indigofera argentea*. SA187 could survive in the rhizosphere as well as in association with different plant species, and was able to provide abiotic stress tolerance to *Arabidopsis thaliana*. The genome sequence of SA187 was obtained by using Pacific BioScience (PacBio) single-molecule sequencing technology, with average coverage of 275X. The genome of SA187 consists of one single 4,429,597 bp chromosome, with an average 56% GC content and 4,347 predicted protein coding DNA sequences (CDS), 153 ncRNA, 7 rRNA, and 84 tRNA. Functional analysis of the SA187 genome revealed a large number of genes involved in uptake and exchange of nutrients, chemotaxis, mobilization and plant colonization. A high number of genes were also found to be involved in survival, defense against oxidative stress and production of antimicrobial compounds and toxins. Moreover, different metabolic pathways were identified that potentially contribute to plant growth promotion. The information encoded in the genome of SA187 reveals the characteristics of a dualistic lifestyle of a bacterium that can adapt to different environments and promote the growth of plants. This information provides a better understanding of the mechanisms involved in plant-microbe interaction and could be further exploited to develop SA187 as a biological agent to improve agricultural practices in marginal and arid lands.

## Introduction

Worldwide agriculture is currently facing big challenges posed by the increase in global population and climate change, and plant growth-promoting bacteria (PGPB) are becoming an important alternative for sustainable crop production (De Zélicourt et al., 2013; Timmusk et al., 2017). The increase in global temperature is drastically affecting the amount of available arable lands, particularly in dryland areas (40% of world land surface), where approximately half of the poorest people live (rural areas in developing countries) (El-Beltagy and Madkour, 2012). Due to climate change, dryland areas (hyper-arid, arid, and semi-arid lands) are expected to rapidly increase, at the same time as crops, cropping systems, rotations and biota will undergo a deep transformation (El-Beltagy and Madkour, 2012).

Plant growth-promoting bacteria are a group of bacteria that are taxonomically unrelated and can establish symbiotic associations with plants to promote their growth under harsh environmental conditions. PGPB can live in the rhizosphere, epiphytically attached to the surface of roots or leaves, or as endophytic bacteria, living inside the plant tissues. PGPB affect plant growth by directly acquiring nutrients (phosphate, nitrogen, iron) or modulating plant hormone levels (auxins, ethylene), and also by indirectly inhibiting pathogenic bacteria (antibiotics) or insects (pesticides) (Glick, 2012).

In order to identify a beneficial strain that might help crops to cope with various environmental challenges, researchers have started to look for halophilic and halotolerant bacteria inhabiting salty and arid ecosystems, which have the potential to promote plant growth under salinity and drought conditions (Jorquera et al., 2012; Mapelli et al., 2013; Liu et al., 2016). In the last decade, the desert has become a niche of growing research interest for bacteria that are adapted to water scarcity, high salinity and high temperature, and could be used to promote growth of crops under these conditions (Goswami et al., 2014; Vurukonda et al., 2016). Desert soils from across the world typically contain a number of ubiquitous bacterial phyla including Actinobacteria, Bacteroidetes, and Proteobacteria (Makhalanyane et al., 2015), and *Halomonas, Salicola*, *Bacillus*, *Paenibacillus*, *Klebsiella*, *Enterobacter*, *Serratia*, *Cellulosimicrobium*, *Ochrobactrum*, and *Pseudomonas* have been identified among the most abundant genera in saline and hypersaline rhizospheric soils (Bhatnagar and Bhatnagar, 2005; Hedi et al., 2009; Köberl et al., 2011; Marasco et al., 2012; Hanna et al., 2013; Köberl et al., 2013; Mapelli et al., 2013; Goswami et al., 2014). Microbes found in desert soils have been subjected to evolutionary adaptation to extreme conditions and show higher abundance of gene functions related to dormancy and stress response than microbes in non-arid environments (Fierer et al., 2012). Moreover, these microbes have been found to enhance soil fertility and promote the growth of plants (Herman et al., 1995; Marasco et al., 2012; Köberl et al., 2013; Goswami et al., 2014).

Following this idea, our group launched the Darwin21 project^1^, with the goal of exploring the microbial diversity of desert pioneer plants and their use in improving agricultural sustainability in dryland and marginal areas. Preliminary results revealed a large diversity of bacteria that have the potential to promote the growth of different crop plants under diverse biotic and abiotic stresses. In the frame of this project, our group also obtained the draft genomes of several PGPB (Lafi et al., 2016a,b,c, 2017a,c).

Among the isolates, one particular endophytic bacterium, SA187, showed the highest consistency in terms of growth promotion among all performed laboratory tests. SA187 was isolated from root nodules of *Indigofera argentea*, an indigenous desert plant growing in the Kingdom of Saudi Arabia. Based on the analysis of the almost complete 16S rRNA gene (>1400 bp), SA187 was classified as member of the family *Enterobacteriaceae* and named as *Enterobacter* sp. SA187 (Lafi et al., 2017b). The family *Enterobacteriaceae* contains a large number of genera that are found in different environmental niches and are biochemically and genetically closely related. Strains belonging to the genus *Enterobacter* have been isolated from the Atacama Desert, northern Chile, showing tolerance to arsenic (Azua-Bustos and González-Silva, 2014), in the desert soil in Algeria showing capability to degrade glyphosate (Benslama and Boulahrouf, 2013, 2016), or in the rhizosphere of desert plants from oil-polluted soils (Diab et al., 2017). Moreover, members of this genus have been found as endophytic plant growth promoters in date palms (*Phoenix dactylifera* L.) under saline conditions or in association with other diverse plants growing in arid lands (Park et al., 2005; Shankar et al., 2011; Yaish et al., 2015). Members of the genus *Enterobacter* have also been reported to show PGP traits, e.g., *Enterobacter* sp. EnB1, isolated from semidesert soil in Mexico, which has been reported to be able to solubilize phosphate (Delgado et al., 2014), or *Enterobacter* sp. B6, isolated from desert soil in Algeria, which showed biopesticide properties against *Locusta migratoria* L5 nymphs (Oulebsir-Mohandkaci et al., 2015).

Over the past few years, a number of *Enterobacter* sp. and close relatives in the family *Enterobacteriaceae* showing PGP under abiotic stress have been also characterized. For example, *Enterobacter cloacae* SBP-8 (formerly *Klebsiella* sp. SBP-8), which induced systemic tolerance in wheat under salt stress (Singh et al., 2015, 2017), *E. cloacae* UW5, which was able to produce high-levels of indole-3-acetic acid (IAA) (Coulson and Patten, 2015), or *E. oryzae* Ola 51^T^, which was capable to fix atmospheric nitrogen (Peng et al., 2009). Additionally, members of the genera *Enterobacter*, *Klebsiella*, and *Leclercia* have also been reported as phosphate-solubilizers, nitrogen-fixers, and producers of antifungal compounds, phytohormones and siderophores (Melo et al., 2016).

SA187 promoted the growth of the model plant *Arabidopsis thaliana* under diverse abiotic stresses such as salinity, drought or high temperature, demonstrating an important potential for application as PGPB to improve abiotic resistance and yield of crops in arid lands. Here we present the analysis of the complete genome sequence and the biochemical characterization of SA187, and highlight different metabolic pathways that potentially contribute to plant growth and stress tolerance.

## Materials and Methods

### Genomic DNA Isolation and PCR Amplification

Genomic DNA was extracted from fresh bacterial cultures by using Qiagen’s DNeasy blood and tissue kit (Qiagen GmbH, Hilden, Germany) and treated with RNAse A (MO BIO Laboratories, Carlsbad, CA, United States). The extracted DNA was further purified by using Mo Bio PowerClean Pro DNA Clean-Up kit (MO BIO Laboratories, Carlsbad, CA, United States) following the manufacturer’s instructions. DNA quality and quantity were assessed by using Nanodrop 2000 (Thermo Fisher Scientific, Wilmington, DE, United States) and Qubit 2.0 (Life Technologies, Invitrogen division, Darmstadt, Germany). The purified DNA was shipped to the sequencing facilities by using the DNAstable^®^ Plus (Biomatrica, San Diego, CA, United States).

(GTG)_5_-rep-PCR amplification was carried out in a total volume of 25 μl as described by Andrés-Barrao et al. (2016) by using the GTG5 primer (Rademaker et al., 1998) (Supplementary Table S1). The amplification of the 16S rRNA gene was carried out in a total volume of 20 μl by using the universal primers 27F and 1429R (Supplementary Table S1): 200 ng DNA template was added to 18 μl of PCR mixture [2X Taq Polymerase Master Mix (Promega), 0.75 μM each primer] by using the following program: A initial denaturation of 95°C for 5 min followed by 35 cycles with steps of 94°C for 30 s, 55°C for 45 s and 72°C for 1 min 30 s, and a final extension of 5 min at 72°C. PCR amplification of the housekeeping genes was carried out in a total volume of 50 μl as follows: 100 ng DNA template was added to 49 μl of PCR mixture [Taq DNA polymerase (Invitrogen), 1.5 μM MgCl_2_, 125 μM dNTPs, 0.2 μM each primer, 1.5 μl DMSO] by using the following program: A initial denaturation of 95°C for 3 min followed by 35 cycles with steps of 95°C for 30 s, 57°C for 45 s and 72°C for 1 min 30 s, and a final extension of 5 min at 72°C. All PCR reactions were carried out in a C1000 Touch Thermal Cycler (Bio-Rad, United States), run in 0.8-1.5% agarose gel electrophoresis and visualized in a ChemiDoc^TM^ MP Imaging System (Bio-Rad, United States).

### Whole Genome Sequencing and Annotation

Prior to sequencing, DNA was size selected to 20 kb by using the BluePippin system (Sage Science, Beverly, MA, United States). Whole genome sequencing was performed by DNA Link Korea (Seoul, South Korea) using the PacBio RS II sequencing platform (Pacific Biosciences, Menlo Park, CA, United States). Large-insert libraries were sequenced in single-molecule real-time (SMRT) sequencing cells by using P6-C4 chemistry. PacBio reads were assembled into one single contig by using the *de novo* Hierarchical Genome Assembly Process (HGAP.2) algorithm with default parameters. Genome annotation was conducted by using the in-house Automatic Annotation of Microbial Genomes (AAMG) pipeline (Alam et al., 2013). Briefly, AAMG annotated the genome by first predicting RNAs and open reading frames (ORFs), followed by BLAST and Interproscan. Ribosomal RNAs (rRNAs), transfer RNAs (tRNAs) and other non-coding RNAs (ncRNAs) were predicted by using RNAmmer, tRNAscan-SE and Infernal software, respectively (Alam et al., 2013). ORFs were predicted by using FragGene Scan (Rho et al., 2010) with the training model specific to complete genomes. ORFs were annotated based on BLAST against UniprotKB and Kyoto Encyclopedia of Genes and Genomes (KEGG) databases. Protein core signatures, domains and associated Gene Ontology (GO) were assigned using InterProscan. Circular chromosome and GC skew were computed by using CGViewer Server (Grant and Stothard, 2008) and GenSkew^2^, respectively. Chromosomes of related bacteria were aligned by progressive MAUVE (Darling et al., 2010). Function and pathway analysis was performed by using BlastKOALA web tool of KEGG database (Kanehisa et al., 2016). Function analysis by Cluster of Orthologous Genes (COG) was done by using WebMGA (Wu et al., 2011). In-house blast searches were performed through INDIGO-Desert v1.1 (Alam et al., 2013). Toxin-antitoxin (T/A) systems were retrieved by using TA finder (Shao et al., 2011). Gene clusters for the biosynthesis of secondary metabolites were identified by using antiSMASH v.4.0.1 (Weber et al., 2015).

### Phylogenetic Analysis

Phylogenetic analyses based on 16S rRNA gene and multi-locus sequence analysis (MLSA) were used to evaluate the taxonomical affiliation of SA187. To construct the 16S and MLSA phylogenetic trees, sequences of individual genes of SA187, along with those from reference strains of the genus *Enterobacter* and other closely related genera retrieved from public databases^3^, were aligned by using ClustalW (Thompson et al., 2002). For MLSA analysis, blunt end alignments of partial sequences were obtained for each gene: 615 bp (*infB*), 742 bp (*gyrB*), 642 bp (*atpD*), 637 bp (*rpoB*). The concatenated *gyrB*-*rpoB*-*atpD*-*infB* sequences were used to construct the Neighbor-Joining phylogenetic tree. Phylogenetic and molecular evolutionary analyses were conducted by using MEGA 6 (Tamura et al., 2013). Accession numbers of the sequences used in this study are shown in Supplementary Table S2. Similarity matrices showing the pairwise percentage identity between 16S rRNA gene sequences and housekeeping genes concatenated sequences, as well as the correspondent distance matrices are shown in Supplementary Tables S3 and S4.

For further whole-genome phylogenetic analysis, we obtained related genome sets initially based on megablast and later additional members of selected genera, so that a resolved species tree could be constructed. We gathered 55 genomes of 12 different genera. Once at least 3–5 genomes were obtained for each genera, we predicted genes using FragGeneScan to carry out clustering of protein coding genes using OrthoMCL. This provided us with a list of clusters common and unique to the analyzed genomes. We separated the core set of gene clusters (1250 genes) and aligned each of these using MAFFT aligner. Alignments were then concatenated and FastTree was used with default parameters to obtain a species tree with bootstrap values. Two-way ANIs calculation was performed on http://enve-omics.ce.gatech.edu/ani/index, considering a minimum length of 700 bp and minimum identity of 70%. The fragment options were set to 1000 bp for window size and 200 bp for step size. Accession numbers of the genome sequences used in this study, together with % similarity and ANI values are shown in Supplementary Table S5 and Figure S4.

### Data Deposition

The whole-genome shotgun project was deposited in NCBI/DDBJ/EMBL database under the accession no CP019113.

### Arabidopsis Salt Stress Resistance Screening

*Arabidopsis thaliana* seeds were surface sterilized by 10 min shaking in 70% EtOH + 0.05% sodium dodecyl sulfate (SDS), then washed 2–3 times in milliQ water and let to dry. Sterilized seeds where stratified for 2 days on half-strength Murashige-Skoog Basal Salt broth pH 5.8 (½MS) (Murashige and Skoog, 1962) (Sigma, Germany) agar plates, at 4°C and darkness, then transferred vertically to a Percival (22°C, 16 h light cycle) for germination. Five-day old seedlings were then transferred to fresh ½MS+100 mM NaCl agar plates (6 seedlings/plate) next to a LB agar plug with (+B, treated) or without (-B, control) SA187. Plant growth was followed for 12 additional days, until the plant roots reached the bottom of the plates. To evaluate the effect of the bacterial treatment in the plant growth, aerial fresh weight (AFW), root fresh weight (RFW), total fresh weight (FW), and lateral root density (LRD) were determined at the end of the experiment.

### Quantitative PCR (RT-qPCR)

To evaluate the gene expression of several key genes identified during SA187 genome analysis, the total RNA of SA187 treated roots (dual samples) were extracted by using the NucleoSpin^®^ RNA Plant kit (Macherey-Nagel), following manufacturer’s instructions. SA187 grown overnight in LB liquid was used as a control. Total RNA of control SA187 was extracted by using the RiboPure^TM^ RNA Purification Kit, bacteria (Ambion), following the manufacturer’s instructions. A total of 1 mg of total RNA from dual and control samples was retrotranscribed by using SuperScript^®^ III First-Strand Synthesis System for RT-PCR (Invitrogen), using random hexamers and following manufacturer’s instructions. Two μl of the obtained cDNA solution diluted 10-fold was mixed with specific primers (Supplementary Table S1) and SsoAdvanced^TM^ Universal SYBR^®^ Green Supermix (Bio-Rad), and the qPCR reactions were performed in a CFX96 Touch^TM^ Real-Time PCR Detection System (Bio-Rad) as follows: 95°C for 3 min, 40x [95°C for 10 s and 60°C for 40 s], 95°C for 10 s and a final melting curve [65–95°C, 0.5°C increment, for 5 s]. All reactions were performed in three biological replicates. Gene expression values were calculated relative to the housekeeping gene *infB*, by using the ΔΔ*C*_t_ method.

### Biochemical Assays

#### Evaluation of Plant Growth Promoting Traits and Tolerance to Abiotic Stresses

Plant growth promoting (PGP) traits were evaluated by using clearing assays. The ability of SA187 to solubilize phosphate was assessed on Pikovskaya’s (PVK) agar plates (M520, Himedia). The production of siderophores was determined by Blue Agar CAS assay, as described by Louden et al. (2011). Zinc solubilization was assessed on modified PVK agar plates, supplemented with 0.1% ZnCO_3_, as described by Bapiri et al. (2013). Assays were performed by inoculating 30 μl of overnight LB bacterial culture into cavities of ∼0.5 cm in diameter. The production of indole-3-acetic acid was qualitatively determined according to Bric et al. (1991), with the following modifications: LB broth supplemented with 2.5 mM L-tryptophan were used in 96-well plates, which were incubated at 28°C and 190 rpm for 2 days. Tolerance to drought and salt stresses were evaluated by growing SA187 in LB broth (Lennox L Broth Base, Invitrogen) supplemented with 20% polyethylene-glycol (PEG) 6000 and 3% or 5% NaCl, respectively. Tolerance to heat stress was assessed by using LB agar plates incubated at 37°C or 42°C. Assays were performed in triplicates and plates and liquid cultures were incubated at 28°C for 2–5 days.

#### Evaluation of Bacterial Growth in Different Carbon Sources and Salt Concentrations

SA187 was grown overnight in LB broth (Lennox L Broth Base, Invitrogen) at 28°C with 190 rpm until the culture reached the exponential growth phase. Cells were harvested by centrifugation, washed twice with 10 mM MgSO_4_, and finally resuspended in 10 mM MgSO_4_ to a final OD_600_ of 0.5. Thirty microliters of this cell suspension were inoculated by triplicate in 96-well plates, in 300 μl of LB supplemented with increasing concentrations of NaCl (0–4 M) and ½MS alone or supplemented with 1% of the following carbon sources: arabinose, fructose, glucose, glycerol, lactose, maltose, raffinose, sucrose, acetic acid, citric acid, or lactic acid. Bacterial growth was monitored by using a Varioskan Flash microplate reader (Thermo Scientific), where the 96-well plates were incubated for 2 days at 28°C and 300 rpm.

#### Evaluation of Antibiotic Resistance

Antibiotic sensitivity tests were evaluated by clearing assay. Hundred μl of overnight SA187 culture were spread on LB agar plates, where antibiotic impregnated disks were then placed (Fischer Scientific). Clearing rings around the disks indicated inhibition of bacterial growth, hence antibiotic sensitivity (S). When SA187 was able to grow normally and no clearing ring was observed, the bacterium contained the corresponding antibiotic resistance marker (R).

## Results and Discussion

### Genome Sequence Assembly and General Features

**FIGURE 1:**
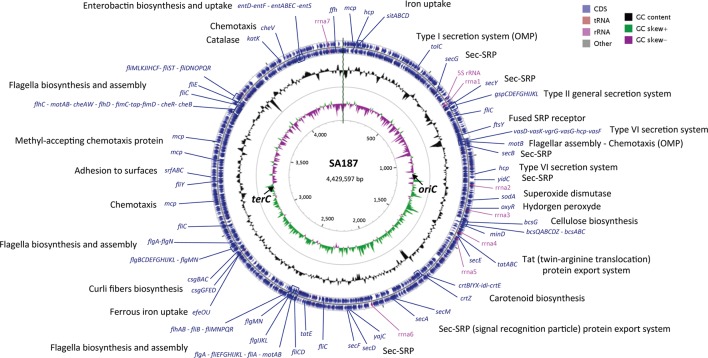
Genome map of the SA187. The bacterial chromosome is 4.4 Mb in size. The outer concentric circles (blue) include the annotation, location and direction of expression of predicted genes, the middle circle (black) indicates the % GC content, and the inner circle indicates de GC skew [(G–C)/(G+C)] positive (green) and negative (purple). A number of interesting genes are highlighted.

Previously, our group published a SA187 draft genome, using Illumina MiSeq technology and obtaining a final assembly of 13 scaffolds (Lafi et al., 2017b). In the present work, PacBio reads were *de novo* assembled by using the Hierarchical Genome Assembly Process (HGAP.2) software and the PBcR pipeline, resulting in 4,793 filtered and preassembled sequence reads with a mean length of 13,471 bp and 275X genome coverage. Consensus polished assembly yielded one circular contig (**Figure 1**). Accordingly, the genome of SA187 consists of a single circular chromosome of 4,429,597 bp, with an average 56% GC content and no plasmids (**Table 1**). A clear GC skew transition was observed, and the origin of replication (*oriC*) and terminus (*terC*) were identified at the positions 1,071,819 and 3,184,452, respectively (**Figure 1**). A total of 4,606 ORFs were identified, including 4,347 protein coding DNA genes (CDS), 153 ncRNAs, 7 complete rRNAs, one additional 5S rRNA, and 84 tRNAs. This number of rRNAs and tRNAs is typical of soil bacteria and an indication of positive selection (Trujillo et al., 2014). Indeed, a high number of rRNAs is a typical characteristic of soil microorganisms, which are able to respond rapidly to changing availability of nutrients (Klappenbach et al., 2000; Shrestha et al., 2007; Lee et al., 2008). Among the CDS, a total of 3,779 (82%) were annotated as genes with a biological function, while 568 (12.3%) were annotated as hypothetical proteins or proteins with unknown function (**Table 1**). 27 ncRNAs were identified as clustered regularly interspaced short palindromic repeat (CRISPR) RNA direct repeat elements, part of the CRISPR/Cas system, which provides acquired resistance against bacteriophages. This result suggests that the genome of SA187 may have been shaped by interaction with bacteriophages (Horvath and Barrangou, 2010; Al-Attar et al., 2011). AntiSMASH analysis revealed 18 clusters for the biosynthesis of secondary metabolites (Supplementary Table S6). Among them, biosynthetic clusters were identified for lipopolysaccharides, emulsan, O- and K-antigens, colonic acid, carotenoids, streptomycin, asukamycin and turnerbactin.

**Table 1:** SA187 genome structure and general features.

Feature	Chromosome
Genome size	4,429,597
GC content	56%
ORF	4606
Gene density	1,039.8 genes/Mb
CDS	4347
– Genes with known function	3779
– Hypothetical proteins	568
Genes assigned to KEGG	2790 (64.2%)
Genes assigned to COG	3574 (82.2%)
ncRNAs	153
– CRISPR RNA direct repeats	27
rRNAs	22
– 16S-23S-5S operons	7
– 5S rRNA	1
tRNAs	84

We performed functional analysis by Cluster of Orthologous Groups (COGs) (**Table 2A** and Supplementary Figure S1A). 3574 out of the 4347 predicted CDSs (82.2%) were assigned to a COG category. This result revealed three main functional gene classes: Carbohydrate transport and metabolism (G), amino acid transport and metabolism (E) and transcription (K), representing 26.85% of the predicted CDS. Another high percentage cluster (20.54%) represented genes involved in energy production and conversion (C), cell wall/membrane/envelope biogenesis (M), inorganic ions transport and metabolism (P) and signal transduction (T). Almost 17% of the predicted CDS are poorly characterized: general function prediction only (R) and with function unknown (S). The high proportion of genes involved in transport and metabolism of carbohydrates, amino acids and inorganic ions, indicate the inherent capacity of SA187 to compete with other microorganisms and survive in the rhizosphere (Niazi et al., 2014). Essential genes from the COG functional categories G and K have been found to have a lower rate of evolution compared with the corresponding non-essential genes (Luo et al., 2015).

**Table 2A:** Functional cluster of orthologous genes (COG) classification of predicted genes in SA187.

COG functional class	CDS	% of CDS
**Metabolism**		
C - Energy production and conversion	220	5.06
E - Amino acid transport and metabolism	372	8.56
F - Nucleotide transport and metabolism	79	1.82
G - Carbohydrate transport and metabolism	473	10.88
H - Coenzyme transport and metabolism	162	3.73
I - Lipid transport and metabolism	101	2.32
P - Inorganic transport and metabolism	220	5.06
Q - Secondary metabolites biosynthesis, transport and catabolism	63	1.45
**Cellular processes and signaling**		
D - Cell cycle control, cell division, chromosome partitioning	34	0.78
M - Cell wall/membrane/envelope biogenesis	229	5.27
N - Cell motility	133	3.06
O - Post-translational modification, protein turnover, chaperones	141	3.24
T - Signal transduction mechanisms	224	5.15
U - Intracellular trafficking, secretion and vesicular transport	112	2.57
V - Defense mechanisms	44	1.01
**Information storage and processing**		
A - RNA processing and modification	1	0.02
J - Translation, ribosomal structure and biogenesis	182	4.19
K - Transcription	322	7.41
L - Replication, recombination and repair	140	3.22
**Poorly characterized**		
R - General function prediction only	399	9.18
S - Function unknown	329	7.57

The functional analysis performed by using the KEGG identified 2790 genes (64.2% of all CDSs) involved in any of the metabolic pathways included in the knowledgebase (**Table 2B** and Supplementary Figure S1B). The analysis revealed the largest number of identified genes as unclassified (10.42%). From those genes that were classified among the KEGG pathway categories, the largest number was involved in metabolism of carbohydrates (7.84%), amino acids (4.23%) and cofactors and vitamins (3.69%). Most of the remaining genes were involved in processes related to environmental and information processing: Membrane transport [ABC transporters 4.88%, phosphotransferase systems (PTS) 2.32%, secretion systems 0.85%] and signal transduction (two-component systems 4.32%). These results confirmed a preference toward metabolism and transport of carbohydrates and amino acids, as well as signal transduction, consistent with the previous results from COG functional analysis.

**Table 2B:** Functional Kyoto Encyclopedia of Genes and Genomes (KEGG) pathway classification of predicted genes in SA187.

KEGG pathway functional class	CDS	% of CDS
**Metabolism**		
– Carbohydrate metabolism	341	7.84
– Lipid metabolism	63	1.45
– Nucleotide metabolism	99	2.28
– Amino acid metabolism	184	4.23
– Metabolism of other amino acids	64	1.47
– Glycan biosynthesis and metabolism	49	1.13
– Metabolism of cofactors and vitamins	168	3.86
– Metabolism of terpenoids and polyketides	44	1.01
– Biosynthesis of other secondary metabolites	27	0.62
– Xenobiotic biodegradation and metabolism	30	0.69
**Genetic information processing**		
– Transcription	159	3.66
– Translation	230	5.29
– Folding, sorting and degradation	52	1.20
– Replication and repair	88	2.02
**Environmental information processing**		
– Membrane transport: ABC Transporters	259	5.96
– Membrane transport: Phosphotransferase system (PTS)	71	1.63
– Membrane transport: Bacterial secretion system	35	0.81
– Signal transduction: Two-component system	119	2.74
**Cellular processes**		
– Cellular community: Quorum sensing	75	1.73
– Cellular community: Biofilm formation	94	2.16
– Cell motility: Bacterial chemotaxis	36	0.83
– Cell motility: Flagellar assembly	72	1.66
**Poorly characterized**		
Unclassified	453	10.42

### Taxonomic Affiliation

**FIGURE 2:**
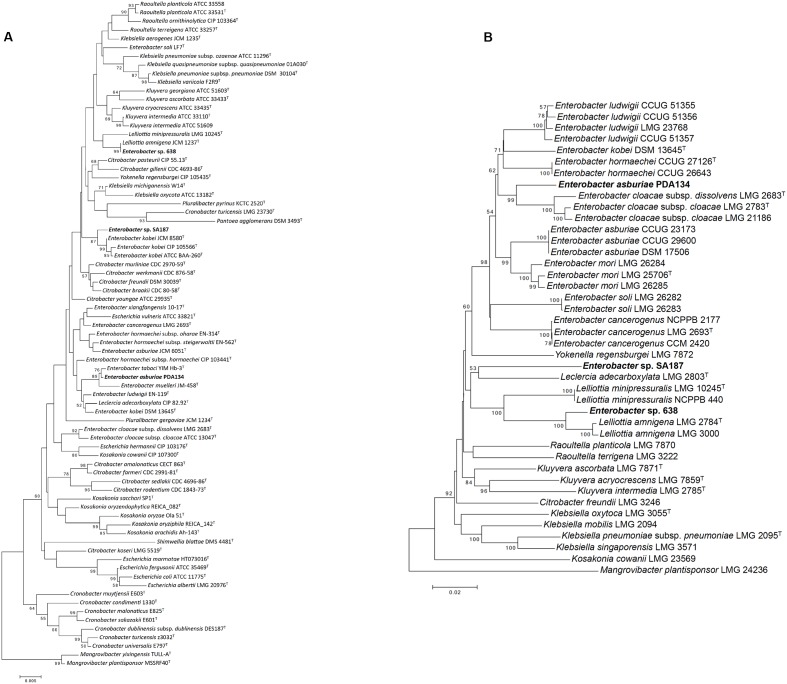
Taxonomic analysis. **(A)** 16S rRNA based phylogenetic tree. **(B)** Multilocus sequence analysis (MLSA) based on four housekeeping genes *gyrB*-*rpoB*-*atpD*-*infB*. The phylogenetic tree was inferred by using the Neighbor-Joining method (Saitou and Nei, 1987), and the optimal tree is shown. The percentage of replicate trees in which the associated taxa clustered together in the bootstrap test (1000 replicates) is shown next to the branches (bootstrap >50 is shown) (Felsenstein, 1985). The tree is drawn to scale, with branch lengths in the same units as those of the evolutionary distances used to infer the phylogenetic tree. The evolutionary distances (number of nucleotide substitutions per site) were computed using the Kimura-2-parameter method (Kimura, 1980). All ambiguous positions were removed for each sequence pair. There was a total of 2,636 positions in the final dataset.

SA187 was first identified as *Enterobacter* sp., closely related to *E. kobei*, based on the full 16S rRNA gene (Lafi et al., 2017b) (**Figure 2A** and Supplementary Tables S3A, S4A). However, based on the 16S rRNA gene alone, the polyphyletic nature of the genus *Enterobacter* makes its classification very difficult. Brady et al. (2008) developed the use of Multilocus Sequence Analysis (MLSA) based on four housekeeping genes (*gyrB*-*rpoB*-*atpD*-*infB*) to obtain a more accurate classification within the family *Enterobacteriaceae*. By applying MLSA, SA187 appeared in a separate clade, distant from the group *Enterobacter* (**Figure 2B**). The taxonomic position of SA187 appeared in a cluster of strains related to the genus *Lelliottia*, with the closest relative *Leclercia adecarboxylata* LMG 2803^T^ (**Figure 2B**). Nevertheless, the low similarity (92%) and the high evolutionary distance (82%) (Supplementary Tables S3B, S4B and Figure S2) between the concatenated sequences of SA187 and LMG 2803^T^, together with the low robustness of the clade (bootstrap 53%) (**Figure 2B**), do not allow the classification of the two strains as members of the same genus. These results suggest that SA187 represents a novel taxon within the family *Enterobacteriaceae*.

Similarly, strains 638 and PDA 134, which were used as reference for comparative genomic analyses, were initially identified based on partial 16S rRNA as *Enterobacter* sp. and *Klebsiella oxytoca*, respectively (Taghavi et al., 2009; Gu et al., 2014), and strain PDA 134 was further reclassified as *E. asburiae* (Yaish, 2016). Unlike this, the position of these strains in the MLSA phylogenetic tree (**Figure 2B**) reveals *Enterobacter* sp. 638 as a new species in the genus *Lelliottia*, and PDA 134 in a different cluster from that formed by the *E. asburiae* group. The evolutionary distance between *E. asburiae* PDA 134 and its closer relatives suggests the strain could represent a new species within the genus *Enterobacter* (**Figure 2B**).

Further whole-genome analysis confirmed these results (Supplementary Figure S3). In this case, SA187 seemed to be closely related to the recently proposed genus *Kosakonia* (Brady et al., 2013), although cannot yet be taxonomically affiliated to any of the closest known species, as ANI values among all the sequences included in the phylogenetic tree ranged 78–81%. Consistently with the MLSA results, *Enterobacter* sp. 638 (Supplementary Table S5 and Figure S4) appeared to belong the genus *Lelliottia*, and *E. asburiae* PDA134 was closely related to the *E. cloacae* group (Supplementary Figure S3).

### Arabidopsis Plant Growth Promotion under Salt Stress

**FIGURE 3:**
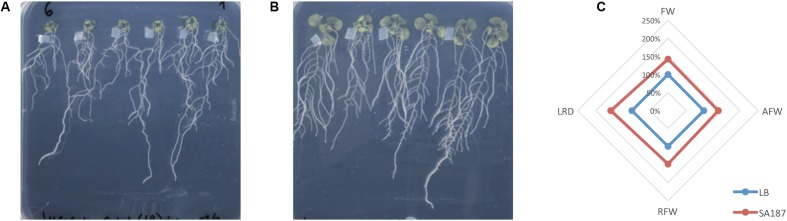
Plant growth promotion under salt stress. **(A)** *Arabidopsis thaliana* growing in ½ MS+100 mM NaCl, with no bacterial treatment (–B, control). **(B)** *A. thaliana* growing in ½ MS+100 mM NaCl, treated with SA187 bacterialized plug (+B). **(C)** Radar chart representing the effect of SA187 treatment in the growth of Arabidopsis. AFW, aerial fresh weight; RFW, root fresh weight; FW, total fresh weight; LRD, lateral root density.

The treatment of *A. thaliana* under salt stress conditions (½MS+100 mM) with SA187 showed a clear PGPB effect. After 12 days growth, seedlings treated with SA187 showed bigger shoots and more developed root systems (**Figures 3A,B**) and an increment of 50% in all measured parameters (**Figure 3C**).

**FIGURE 4:**
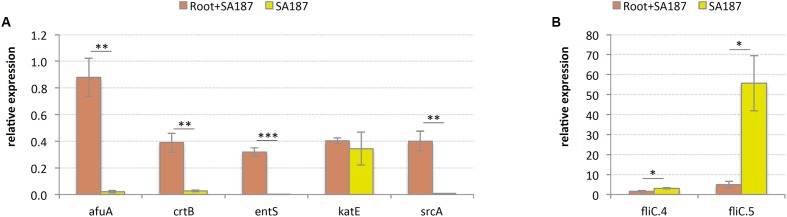
Relative gene expression by RT-qPCR. **(A)** SA187 genes increasing their expression upon association with Arabidopsis roots. *afuA* = iron(III) ABC transporter substrate-binding protein, *crtB* = phytoene synthase, *entS* = MFS transporter ENTS family enterobactin (siderophore) exporter, *katE* = catalase, *srcA* = PTS system, sucrose-specific IIB component. **(B)** SA187 genes decreasing their expression upon association with Arabidopsis roots. *fliC* = flagellin. ^∗^ = significant (*p* < 0.05), ^∗∗^ = very significant (*p* < 0.01), ^∗∗∗^ = extremely significant (*p* < 0.001).

Gene expression of several key genes later identified in this report, gave hints to elucidate a possible mechanism for plant growth promotion: the gene coding for iron(III) ABC transporter substrate-binding protein, *afuA*; phytoene synthase, *crtB*; MFS transporter ENTS family enterobactin (siderophore) exporter, *entS*; and PTS system, sucrose-specific IIB component, *srcA*, were highly expressed when SA187 was associated with roots, compared with the pure bacterial culture, suggesting their role in plant growth promotion (**Figure 4A**). The gene coding for catalase, *katE*, did not show significant differences (**Figure 4A**), and 2 genes coding for the protein flagellin, *fliC*, a structural part of the bacterial flagellum, decreased its expression upon association with Arabidopsis roots (**Figure 4B**). The decrease in expression of *fliC* genes is consistent with the transition from a highly aerated shacked liquid culture to a situation where SA187 has stably colonized the plant root.

### Biochemical Characteristics of SA187

The qualitative evaluation of PGP traits showed that SA187 was able to produce siderophores and also to solubilize zinc, but was unable to solubilize phosphate (**Table 3**). These results revealed some of the possible strategies that the bacterium might employ when interacting with its host plant, contributing to plant growth promotion. The obtained antibiogram showed that SA187 is resistant to both ampicillin and penicillin G, but sensitive to chloramphenicol, erythromycin, kanamycin, oxytetracycline, streptomycin, tetracycline and rifampicin (**Table 4**). The resistance to ampicillin and penicillin G is providing SA187 with an additional strategy to compete with other coexisting bacteria and fungi in the desert soil and in the rhizosphere.

**Table 3:** Biochemical characteristics of SA187.

Plant growth promoting (PGP) traits	
Phosphate solubilization	-
Zinc solubilization	+
Siderophore production	+

*- = no clearing zone was observed on agar plates, + = clearing zone appeared after 24 h incubation*.

**Table 4:** Resistance of SA187 to different antibiotics.

Antimicrobial compound	
Ampicillin	R
Chloramphenicol	S
Erythromycin	S
Kanamycin	S
Oxytetracycline	S
Penicillin G	R
Streptomycin	S
Tetracycline	S
Rifampicin	S

*R = bacterial growth was observed, indicating resistance to the antibiotic, S = no bacterial growth was observed, indicating sensitivity to the antibiotic*.

SA187 grew well under osmotic and heat stress conditions, as well as under salt stress, up to a concentration of 1 M NaCl (**Table 5**). When comparing the growth of the bacterium under increasing concentrations of NaCl, we observed that supplementing LB broth with low concentrations of NaCl (0.1–0.5 M) had a beneficial effect on bacterial growth. The maximum cell density in these cases (OD_600_), when compared with LB with no additional NaCl added, increased about 0.2 units. The growth of SA187 was in contrast slightly delayed when 1 M NaCl was added, and completely inhibited at higher concentrations (LB+2 M NaCl and LB+4 M NaCl) (**Table 5** and Supplementary Figure S5A). These results indicate that SA187 is moderately halophilic, being able to resist NaCl concentrations up to 1 M. Similar results were recently reported for *Klebsiella* sp. BRL6-2, which was able to grow under salt conditions up to 1.5 M NaCl (Woo et al., 2014). The capability to resist moderate salt stress would contribute to the survival of SA187 as a free-living bacterium in desert soils.

**Table 5:** Resistance of SA187 to different abiotic stresses.

Resistance to abiotic stresses	
**Salt stress**	
– Growth LB^∗^ (no NaCl added)	+
– Growth LB + 0.1 M NaCl	+
– Growth LB + 0.25 M NaCl	+
– Growth LB + 0.5 M NaCl	+
– Growth LB + 1.0 M NaCl	+
– Growth LB + 1.5 M NaCl	-
– Growth LB + 2.0 M NaCl	-
**Osmotic stress**	
– Growth in 20% PEG 6000	+
**Heat stress**	
– Growth at37°C	+
– Growth at 42°C	+

*- = no bacterial growth was observed, + = positive bacterial growth was observed. ^∗^normal pH of LB medium was adjusted to 7.0*.

The evaluation of the growth of SA187 in different carbon sources showed that the bacterium grows fast in LB broth (containing 0.5% NaCl), as expected for this nutrient rich medium. Consistently with the lack of a utilizable carbon source in ½MS, SA187 did not show any growth when no sugar was added to this medium. SA187 did not grow in ½MS supplemented with 1% acetic acid or 1% lactic acid. SA187 did nevertheless grow in ½MS supplemented with 0.1–1% sucrose, 1% arabinose or 1% citric acid, although the log phase was clearly delayed (>12 h) compared to LB (Supplementary Figure S5B). SA187 was also able to grow in ½MS+1% glycerol and ½ MS+1% lactose to some extent, although the growth rates in these cases were strongly reduced. Finally, compared with ½MS the growth of SA187 was slightly stimulated when 1% fructose, 1% raffinose, 1% glucose, or 1% maltose were added (Supplementary Figure S5B).

Additionally, the pH of the culture broth has a clear influence on the bacterial growth. The good growth in LB compared with all other conditions might be also due to the higher pH to which the medium is adjusted (pH 7.0), compared with the pH in ½ MS (pH 5.8). Interestingly, no big difference was observed when SA187 was grown in LB pH 7.0 or LB pH 5.8 (Supplementary Figure S5B), indicating the bacterium is capable to resist certain acid stress due to a lower pH.

### Metabolic Features of SA187 Involved in the Dual Life-Style: Free-Living and Plant-Associated

**FIGURE 5:**
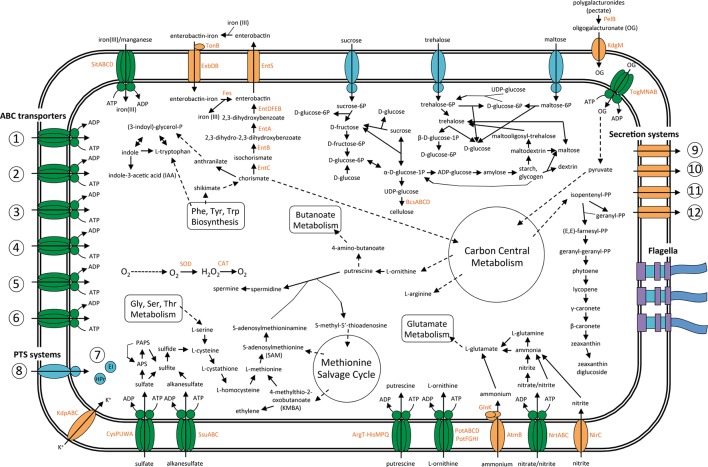
Overview of the metabolism and transport in endophytic bacterium SA187. Predicted pathways involved in plant–microbe interaction, based on the current annotation of the complete genome sequence SA187. Metabolic flow through each pathway is designated by the direction of the arrow connecting two metabolites. Import or export of solutes is designated by the direction of the arrow through the transporter. ABC transporters: (1) Mineral and organic ion transporters: Sulfate, molybdate, nitrate/nitrite, taurine, sulfonate, iron(III), thiamine, spermidine/putrescine, betaine/proline, osmoprotectant; (2) Saccharide, polyol, lipids transporters: Maltose/maltodextrine, L-arabinose, oligogalacturonide, methyl-galactoside, D-xylose, autoinducer-2 (AI-2), rhamnose, ribose, glycerol-3-phosphate, phospholipid, multiple sugar, simple sugar; (3) Phosphate and amino acid transporters: Phosphate, histidine, glutamine, arginine, glutamate/aspartate, general L-amino acid, cysteine, branched-chain amino acid, urea, D-methionine, polar amino acid; (4) Peptide and nickel transporters: Oligopeptide, dipeptide, cationic peptide, nickel, glutathione, peptide/nickel, microcin C; (5) Metallic cation, iron-siderophore, vitamin B12 transporters: Iron-complex transporter, vitamin B12, zinc, manganese/iron; (6) ABC-2 type and other transporters: lipopolysaccharide, lipoprotein-releasing, putative ABC, ABC-2 type. Phosphotranspherase systems (PTS): (7) Enzyme I and phosphocarrier protein (HPr); (8) Enzyme II: Glucose, *N*-acetyl-glucosamine, maltose/glucose, maltose, sucrose, beta-glucoside, trehalose, alpha-glucoside, fructose, mannitol, 2-*O-A*-mannosyl-glycerate, cellobiose, glucitol/sorbitol, galactitol, mannose, fructoselysine, ascorbate. Secretion systems: (9) General (Sec-dependent) secretion system, (10) Tat-dependent secretion system, (11) type 2 secretion system (T2SS), (12) type 6 secretion system (T6SS).

A general overview of the main metabolic pathways and transport systems involved in the interactions between SA187 and its plant-host is presented in **Figure 5**.

#### Survival under Extreme Conditions

SA187 was isolated from *I. argentea*, an indigenous desert plant growing in areas where heat, drought and salt are key environmental stresses with which bacteria must cope during their free-living lifestyle in the soil. Betaine (also called glycine-betaine) and proline are known to confer salt tolerance to organisms (Ren et al., 2010; Hayat et al., 2012). P-blast genome mining revealed that SA187 contains the complete pathway for the biosynthesis of proline. However, the genes coding for choline dehydrogenase (*betA*) and betaine dehydrogenase (*betB*) are not present, indicating that SA187 is unable to synthesize betaine. On the other hand, we found a set of genes coding for membrane transporters: ATP-binding cassette (ABC) transporters ProVWX (SA187PBcda_000000076-000000079) and OpuABCD (SA187PBcda_000004092-000004095), and 3 copies of an major facilitator superfamily (MFS) transporter metabolite:H+ symporters (MHS) family, ProP (SA187PBcda_000002035, SA187PBcda_000002396, SA187PBcda_000004343), which can be used to internalize these osmoprotectants, which might be released into the rhizosphere by other microorganisms and plants. A novel role for ProP as carnitine uptake system has been recently described in *Cronobacter sakazakii* BAA-894 (member of the family *Enterobacteriaceae*). The uptake of carnitine by ProP provided the strain with a higher osmotolerance, being able to grow under salt concentrations far in excess of that afforded by proline (Feeney and Sleator, 2015).

Trehalose is another important osmoprotectant produced under environmental stresses. Five pathways for the biosynthesis of trehalose have been described: TreS, OtsA/OtsB, TreP, TreT, TreY/TreZ (Garg et al., 2002; Paul et al., 2008). In the genome of SA187 we found genes coding for trehalose 6-phosphate synthase and trehalose 6-phosphate phosphatase (*otsAB*, SA187PBcda_000004793-000004794), 2 copies of trehalose-6-phosphate hydrolase (*treC*, SA187PBcda_000000633, SA187PBcda_000002209), malto-oligosyltrehalose trehalohydrolase (*treZ*, SA187PBcda_000002137) and the transcriptional regulator *treR* (SA187PBcda_000002211). Additionally, we found 2 copies of *treA* (SA187PBcda_000001180, SA187PBcda_000004582) coding for the enzyme trehalase, which catalyzes the hydrolysis of trehalose into glucose, as well as 2 copies of *treB* (SA187PBcda_000002210, SA187PBcda_000002946) a PTS that is specific for the uptake of trehalose. The role of trehalose in osmotolerance has been reported recently in *Klebsiella* sp. BRL6-2. The growth rate of this strain in 6% NaCl containing medium supplemented with trehalose increased significantly when compared with the growth in the absence the of osmoprotectants (Woo et al., 2014).

A recent study reported an ATP-dependent potassium (K^+^) uptake system (KdpFABC) to be essential for survival of *Halobacterium salinarum*, an extreme halophilic Gram-negative archeon, under desiccation and high salinity (Kixmüller and Greie, 2012). Homologs of *kdpFABC* are widely distributed among the family *Enterobacteriaceae* and other Gram-negative bacteria and cyanobacteria and have been reported to increase their expression in response to salinity (Walderhaug et al., 1989; Jung et al., 1997; Solheim et al., 2014). One of the prominent responses of *Salmonella enterica* to high ion concentrations has been reported to be the transcriptional induction by more than 100-fold of 2 operons: *proU* (*proVWX*), and the *kdpABC* system (Balaji et al., 2005). The genome of SA187 contains the genes *kdp* forming an operon that also included the genes for the corresponding two-component system response regulator (KdpED) (*kdpEDCBAF*, SA187PBcda_000003311-000003316).

Other mechanisms conferring salt tolerance to halophiles is the presence of cation transport systems for the controlled uptake of sodium (Na^+^), potassium (K^+^) and chloride (Cl^-^) (Flowers et al., 2015). The genome of SA187 contains genes coding for cation/proton (H^+^) antiporters that contribute to osmoregulation: K^+^/H^+^ antiporter NhaP2 (SA187PBcda_000004591) and Na^+^/H^+^ antiporter NhaA (SA187PBcda_000002407). We also found a transcriptional regulator of the family LysR, activator of NhaA (*nhaR*, SA187PBcda_000002407-000002408). The NhaP2 and NhaA transport systems allow bacteria to import H^+^ while pumping K^+^ and Na^+^, thus preventing excessive cation accumulation, and have been recently found to resist hyperosmotic stress in the genome of the alkalotolerant plant growth-promoting rhizobacterium *Klebsiella* sp. D5A (Liu et al., 2016).

**FIGURE 6:**
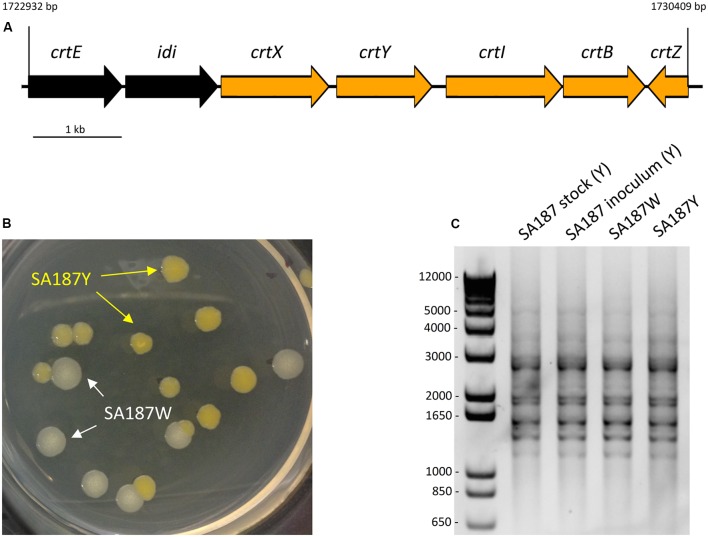
**(A)** Carotenoid biosynthesis gene cluster. **(B)** SA187 multi-phenotypic complex. After a certain period of interaction between SA187 and the plant host Arabidopsis, 2 morphologies differing in the pigmentation are observed: yellow (SA187Y) and white (SA187W). **(C)** (GTG)_5_-rep-PCR fingerprinting. Genotypic characterization of the SA187Y and SA187W isolates, comparison with the original SA187 stock and the inoculum used for one of the screening experiments. The same amplification pattern shown by the four samples shown in the panel indicates that both phenotypes, yellow and white, are genetically identical.

In addition to the biosynthesis of different osmoprotectants, the production of carotenoids has also been reported to be important for the survival of the bacteria in the rhizosphere and its protection against UV radiation, as well as for the bacterial-plant association (Mohammadi et al., 2012; Bible et al., 2016). On synthetic media, SA187 produced a yellow pigment that could be due to the biosynthesis of carotenoids. We found that the genome of SA187 contains all 7 genes of the entire carotenoid biosynthesis pathway, which is organized in a gene cluster (*crtE-idi-crtXYIBZ*, SA187PBcda_000002248-000002254) (**Figure 6A**) that is syntenic with the cluster identified in *C. sakazakii* BAA894, a close relative of the genus *Enterobacter* (Zhang et al., 2014). This *Cronobacter* strain also forms yellow colonies when grown on agar plates, due to the production of two carotenoids: zeaxanthin-monoglycoside and zeaxanthin-diglucoside (Zhang et al., 2014). The conserved synteny of the gene cluster present in SA187 and the one identified in *C. sakazakii* BAA894 suggest that the yellow pigment produced by SA187 could be due to the same zeaxanthins. The importance of carotenoids for an effective plant–microbe interaction was revealed by *Pantoea* sp. YR343 Δ*crtB* mutant. This mutant strain was defective in the production of carotenoids, due to the lack of phytoene synthase (crtB), a homolog of this gene is found in SA187, and was reported to show increased sensitivity to oxidative stress, impaired biofilm formation and production of IAA, and the reduced colonization of plant roots (Bible et al., 2016). By homology with the same genes in *C. sakazakii* BAA894 and other *Enterobacteriaceae* strains, including *Pantoea* sp. (Misawa et al., 1990; Sedkova et al., 2005; Conlan et al., 2014; Zhang et al., 2014), we hypothesized that the yellow pigment produced by SA187 could be a derivate of the carotenoid zeaxanthin, and could have a role in Arabidopsis root colonization. Additionally, zeaxanthin is a precursor in the plant biosynthetic pathway to produce salicylic acid (SA), a plant hormone that could have a role in the PGP provided by SA187.

Two-component systems (TCS) are signaling pathways that allow bacteria to sense and respond rapidly to changes in their environment. TCS consist of a sensor membrane-bound histidine kinase (HK) and a corresponding response regulator (RR) (Mitrophanov and Groisman, 2008). In SA187, a high number of genes are involved in TCS systems and signal transduction (Supplementary Table S7). Most of the SA187 TCS belong to the OmpR family, but also systems belonging to the NarL and NtrC families were identified. Among them, the KdpD/KdpE system (mentioned before), one of the most distributed HK/RR systems in bacteria, which is typically activated under K^+^ limitation or osmotic stress (Heermann and Jung, 2012). We also found the CpxA/CpxR TCS, which controls the envelope stress response in Gram-negative bacteria (*cpxAR*, SA187PBcda_000001596-000001597). In *Escherichia coli*, the CpxA/CpxR system, jointly with the sigmaE and sigma32 response pathways, regulates gene expression in response to adverse conditions (De Wulf and Lin, 2000). A large number of TCS genes is typical for bacteria living in rapidly changing or diverse environments (Capra and Laub, 2012), and correlates with the dualistic life style of SA187 as free-living and plant-associated microorganism.

#### Transport and Exchange of Nutrients

Bacteria living in endophytic association need to exchange nutrients (Chibucos and Tyler, 2009). Consistently, we found that SA187 codes for a large diversity of transporters to allow the exchange of bacterial metabolites and plant-produced nutrients. Among these transporters, we identified more than 200 genes coding for ABC transporters, which among other things, are involved in the uptake of metals (iron, manganese, nickel, molybdate, zinc), phosphate, sulphate, nitrate/nitrite, urea, sugars (glycerol-3P, ribose, rhamnose, xylose, maltose/maltodextrine, arabinose), amino acids (glycine-betaine/proline, methionine, cysteine, arginine, branched-chain amino acids, glutamine/aspartate, histidine, lysine/arginine/ornithine) polyamines (spermidine/putrescine), or quorum sensing autoinducer-2 (AI-2) (Supplementary Table S8). AI-2 and LuxS, the enzyme that catalyzes the production of the signal precursor for AI-2 mediated quorum sensing, has been reported in *Enterobacteriaceae* of the genera *Enterobacter*, *Klebsiella*, and *Pantoea* that live in close association with plants (Rezzonico et al., 2012).

SA187 is also able to incorporate a wide plethora of sugars through PTS. We identified 101 genes involved in the uptake of glucitol/sorbitol, lactose/cellobiose, galactitol, mannitol, fructose, ascorbate, trehalose, or mannose, among others (Supplementary Table S9). We also found 53 genes coding for members of the MFS transporters, such as the MHS family proline/betaine transporter ProP (mentioned before) (Supplementary Table S10). These results are consistent with the capacity of SA187 to grow on different carbon sources, as described previously. SA187 was able to grow in media with arabinose and, to some extent, with glycerol or lactose. SA187 is also able to incorporate sucrose, fructose, glucose or maltose, what is consistent with its growth on ½MS when the corresponding carbon sources were added (Supplementary Figure S5B).

Additionally, bacteria have developed several specific mechanisms to compete for iron, an essential element whose availability often limits bacterial growth. These mechanisms include specific iron uptake transporters, the secretion of large numbers of diverse siderophores and the synthesis of siderophore receptors to utilize siderophores produced by other microorganisms. The presence of an efficient iron uptake system can also contribute to protect the host plant against pathogen infections, by depriving iron from the pathogenic microorganisms (Taghavi et al., 2010). We found 54 genes involved in iron- and manganese-uptake by SA187 (Supplementary Table S11). Among them, several iron ABC transporters: SitABCD, 2 AfuABC, 4 FhuDBC as well as six genes coding for the iron complex-outer membrane receptor FhuA; two ferrous iron uptake transporters: FeoABC and EfeUO; and the ferrous-ion efflux pump FieF (Supplementary Table S11). An important role of iron uptake in the interaction between SA187 and the plant is supported by the observed increase in gene expression of the genes *afuA* and *entS* (**Figure 4A**). These transporters for iron uptake are common among members of the *Enterobacteriaceae* family, including PGPR *Enterobacter* sp. 638 and *Klebsiella* sp. D5A (Boyer et al., 2002; Taghavi et al., 2010; Liu et al., 2016).

Similarly to other *Enterobacteriaceae* (Taghavi et al., 2010; Carpenter and Payne, 2014), SA187 contains the genes necessary to synthesize the siderophore (ferric chelator) enterobactin: *entD* (SA187PBcda_000005352), *entF* (SA187PBcda_000005348), *entABEC* (SA187PBcda_000005338-000005341), to secrete it through an MFS transporter (*entS*, SA187PBcda_000005343), and to recover the enterobactin-iron complex through the TonB-dependent transporter ExbDB (SA187PBcda_000000488-000000489). Finally, the enterobactin esterase Fes (SA187PBcda_ 000005350) will liberate the iron molecule. An additional TonB-dependent outer membrane iron-enterobactin/colicin (*fepA*, SA187PBcda_000005351) was also identified. The production of enterobactin has been reported in *E. coli* BW25113 and *E. cloacae* ATCC 13047, were it has been found to be positively regulated by the peroxiredoxin AhpC (Ma and Payne, 2012; Carpenter and Payne, 2014).

Phosphorus (P) is another element that is an essential macronutrient for the growth of all biota, including plants, and, together with nitrogen, it is one of the major limiting macronutrients for crop production (Bergkemper et al., 2016). Plants are only able to take up free orthophosphate (PO_4_^3-^), but phosphate in the soil is mostly present in the form of insoluble compounds. Therefore, specialized microorganisms such as PGPB play an important role in providing available inorganic P to the plant, in the form of PO_4_^3-^ (Bergkemper et al., 2016). In most bacteria, a mineral phosphate-dissolving capacity has been shown to be related to the production of organic acids, and the direct oxidation of glucose to gluconic acid (GA) has been proposed as the main mechanism for mineral phosphate solubilization in Gram-negative bacteria. This oxidation is carried out by the glucose dehydrogenase (GDH) enzyme and the cofactor pyrroloquinoline quinone (PQQ) and has been identified, among others, in the phosphate solubilizers *E. asburiae* and *Leclercia* sp. QAU-66 (Gyaneshwar et al., 1999; Rodríguez et al., 2007; Naveed et al., 2014). The genome of SA187 contains only the gene coding for PqqF. The lack of the operon *pqqABCDE* eliminates this pathway as the strategy used for phosphate solubilization. Nevertheless, we found that SA187 can synthetize 2 exopolyphosphatases (*ppx-gppA*, SA187PBcda_000001262, SA187PBcda_000005395) and an inorganic pyrophosphatase (*ppa*, SA187PBcda_000002182), which are three enzymes that have been shown to be involved in making insoluble phosphorus available for to plants (Bhattacharyya et al., 2017). The genome of SA187 contains genes coding for the TCS PhoB/PhoR involved in the phosphate starvation response (*phoBR*, SA187PBcda_000002805-000002806) and an ABC transporter for phosphate uptake (*pstSCAB*, SA187PBcda_000001427- SA187PBcda_000001430). Moreover, we also identified a low-affinity inorganic phosphate transporter (*pit*, SA187PBcda_000001153). The genes *phoA* and *phoD*, coding for enzymes alkaline phosphatases, which release PO_4_^3-^ and acts downstream the PhoBR system, were not found. The Pst transporter is repressed by phosphate and induced under phosphate limitation, while the Pit system is constitutive (Jansson, 1988). Despite the potential capability of SA187 to incorporate phosphate (PO_4_^3-^) through these systems, the bacterium was unable to experimentally solubilize phosphate, as shown by our biochemical analysis (**Table 3**), suggesting that the transporters might be non-functional or not expressed under the experimental conditions.

Together with P and K^+^, nitrogen is one of the most important micronutrients for the plant. A number of *Enterobacteriaceae*, including *Enterobacter oryzae* Ola51^T^, *E. agglomerans, E. cloacae*, or *L. adecarboxylata* STUPM20, have been reported to be nitrogen-fixers (Kreutzer et al., 1991; Peng et al., 2009; Laili et al., 2017). Interestingly, the genome of SA187 lacks genes coding for the nitrogenase enzyme (*nifDHK)*, required for nitrogen fixation, but contains genes for dissimilatory nitrate reduction (Supplementary Table S12): *narLXK* (SA187PBcda_000004490-000004493), *narGHIJ* (SA187PBcda_000004486-000004489), *nirBD* (SA187PBcda_000001018-000001019), *nirC* (SA187PBcda_000001020) and nitrate assimilation: *nasAB* (SA187PBcda_000004496-000004497). We also found an ammonium uptake transporter and its regulator (*amtB* and *glnK*, SA187PBcda_000002879-000002880), a periplasmic nitrate reductase (*napA*, SA187PBcda_000005261), a nitrate/nitrite ABC transporter (*nasDEF/nrtABC*, SA187PBcda_000004498-000004500) and the nitrate RR NasR (SA187PBcda_000004502). These results indicate that SA187 is potentially able to incorporate nitrate and nitrite for assimilation into ammonia, as well as to incorporate ammonia directly.

#### Secretion of Effector Proteins

Besides the uptake and exchange of nutrients, bacteria also need a set of different protein secretion systems that are essential for their growth and for their interaction with plants. Through these systems, bacteria secrete enzymes, peptides, toxins, antibiotics or secondary metabolites to the surrounding environment, to compete with nearby microorganisms or to be incorporated and used by their host plant (Green and Mecsas, 2016). Among the bacterial secretion systems, the general secretion (Sec) and the twin-arginine translocation (Tat) pathways are most commonly used to transport proteins across the plasma membrane (Natale et al., 2008). The Sec pathway primarily secretes unfolded proteins, while the Tat pathway is mostly used to secrete folded proteins (Robinson and Bolhuis, 2004). Most of the proteins transported by these pathways remain inside the cell, but in Gram-negative bacteria, they can either stay in the periplasm or the inner membrane, or they can be secreted outside through the type II (T2SS) or type V (T5SS) secretion systems (Green and Mecsas, 2016). In the genome of SA187, we identified 35 genes involved in bacterial secretion systems. Among them, we identified a complete Sec and Tat secretion pathways and most of the T2SS and type VI (T6SS) secretion systems (Supplementary Table S13). We did not find any of the genes required for the biosynthesis of type III (T3SS) nor T5SS.

The T2SS is conserved in most Gram-negative bacteria, and is unique in its ability to promote secretion of large and sometimes multimeric proteins that are folded in the periplasm and transport them into the extracellular environment (Douzi et al., 2012; Green and Mecsas, 2016). Although T2SS has been found in many plant pathogens such as *Pseudomonas fluorescens*, *Erwinia*, or *Xanthomonas* spp., T2SS is also important for non-pathogenic bacteria, such as the metal reducing bacteria *Shewanella oneidensis* (Douzi et al., 2012; Korotkov et al., 2013). In the genome of SA187 we found almost all core genes coding for the T2SS, which were organized in an operon (*gspCDEFGHIJKLM*, SA187PBcda_000000965-000000975). Only the genes *gspO*, coding for a prepilin peptidase, and *gspS*, coding for an accessory pilotin, were not found. As non-core component, genes encoding GspS have not been found in all bacterial species. Nevertheless, the T2SS core protein GspO seems to be essential for the secretion system to be functional (Douzi et al., 2012). Although no gene coding for GspO was found in the genome of SA187, we found two genes showing a high homology, coding for a type IV prepilin-like proteins peptidase (*pilD*, SA187PBcda_000000976, SA187PBcda_000000320), which could be used instead, rendering SA187 with a fully functional T2SS secretion system.

The T6SS, on the other hand, is the most recent bacterial secretion system to be discovered and also fairly well conserved among Gram-negative bacteria. Although it is still poorly characterized, T6SS translocates effector proteins into a variety of recipient cells, including eukaryotic cells and other bacteria and has been reported in a well-studied PGPB strain *P. fluorescens* (Decoin et al., 2014; Green and Mecsas, 2016). Many of these effectors are directed against the bacterial cell wall and membrane, supporting a role in bacterial competition with other microorganisms (Russell et al., 2011, 2014). Similarly to T2SS, T6SS has been found in pathogenic as well as in non-pathogenic bacteria (Shyntum et al., 2014). In the case of SA187, we identified most of the genes coding for T6SS, including three copies of the gene coding for the Hcp protein, (SA187PBcda_000000063, SA187PBcda_000001145, SA187PBcda_000001383), but none of the three post-translational regulators: PpkA, Fha1 and Stp1. These regulatory proteins belong to the non-core set of genes and are not essential for the biosynthesis of a functional secretion system (Shyntum et al., 2014).

#### Chemotaxis and Bacterial Mobility

Motility is an important characteristic for plant-associated bacteria and endophytes, enabling bacteria to move and colonize plants and also to systematically spread within the plant (Hardoim et al., 2008). We identified 156 genes involved in chemotaxis and biosynthesis and assembly of flagella (Supplementary Table S14). The most widespread bacterial chemotaxis signaling pathway centers on a fixed core of signaling genes, consisting in the TCS CheA/CheY, methyl-accepting chemoreceptor proteins (MCP) and an adaptor protein CheW (Capra and Laub, 2012). Consistently with its chemotactic nature, we found that SA187 is able to synthetize the TCS CheA/CheY (*cheA*, SA187PBcda_000004788; *cheY*, SA187PBcda_000004777), as well as CheW (SA187PBcda_000004787) and a wide variety of MCPs (10 *mcp*, *tap*, 3 *tar*, 2 *tgr*, 4 *tsr*) (Supplementary Tables S6, S14). We also found genes coding for additional chemotaxis proteins: CheZYBR (SA187PBcda_000004776-000004779), CheWA (SA187PBcda_000004787-000004788), the TCS RR CheV (SA187PBcda_000005252) and CheB/CheR fusion protein (*cheBR*, SA187PBcda_000000470), and also several copies of the genes coding for fimbrial proteins FimA, FimC, and FimD (Supplementary Table S14).

The mobile nature of SA187, which allows the bacterium to move through the soil matrix and inside the plant, was confirmed by the presence of a large number of genes involved in the biosynthesis and assembly of flagella, such as 2 operons *flgABCDEFGHIJKLMN*, 2 *flhAB*, *flhCD*, *flhE*, 2 *fliA*, *fliB*, 2 *fliCD*, 3 *fliC*, *fliEFGHIJKLMN*, *fliEFGHIJKLMNOPQRST*, as well as and 2 copies of genes coding for the flagellar motor proteins MotA and MotB. We also found genes involved in the biosynthesis and assembly of the type IV pilus system (T4PS) (*hofBC*, *hofMNOPQ*, *tcpC*, *tcpD*, *tcpE, tcpT*) and pilin (2 *pilD*, *ppdABC*, *tcpA*, *tcpB*) (Supplementary Table S14).

**FIGURE 7:**
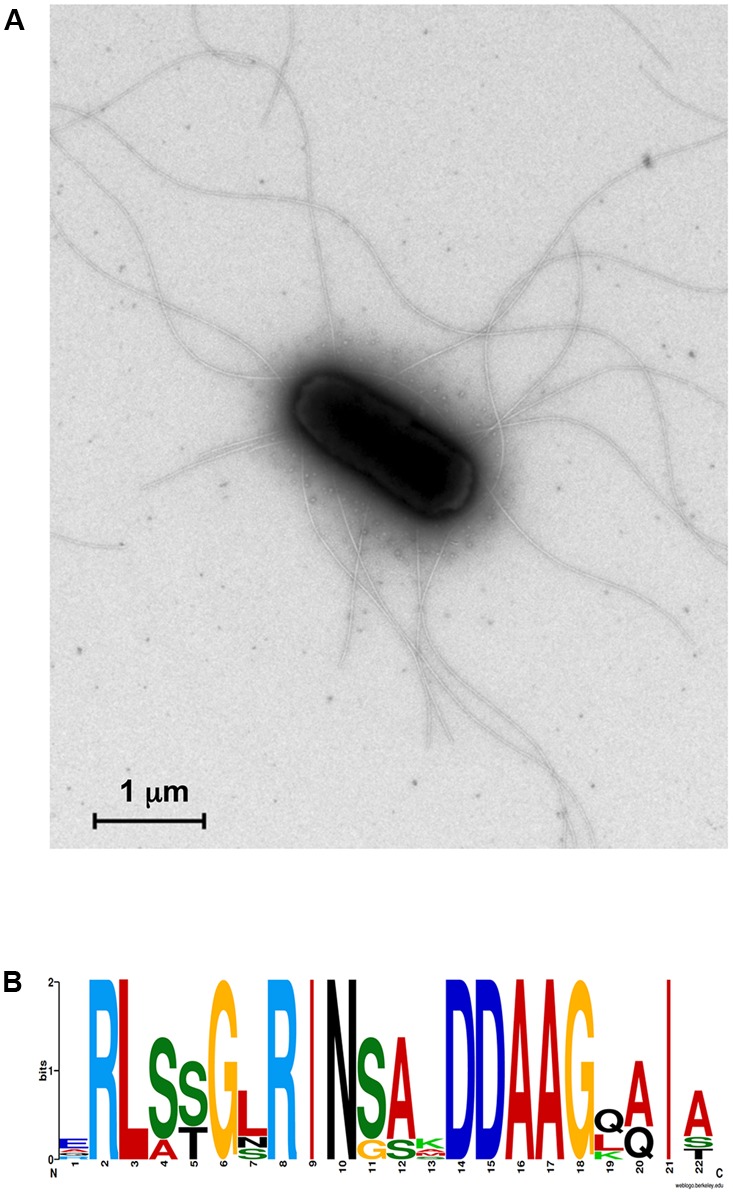
**(A)** Transmission electron microscopy section of SA187. Bacteria were cultured in LB broth before fixation and negative staining. SA187 shows a high number of peritrichous flagella. **(B)** Conservation of flg22 motif. The N-terminal of FliC proteins of SA187 shown a highly conserved motif shared with *Pseudomonas* flg22. Diagram obtained by using WebLogo on-line tool (Crooks et al., 2004).

It is known that T2SS and T4PS are evolutionary related and shared several structural and functional features, such as the prepilin peptidase PilD, as mentioned before (Korotkov et al., 2013). Interestingly, we observed that, in the genome of SA187, most of the genes involved in the biosynthesis of flagella, as well as a set of genes coding for T4PS are grouped in clusters (**Figure 1**). The high number of flagella synthetized by SA187 can be seen in the negative-stained transmission electron microscopy sections shown in **Figure 7A**. Interestingly, 5 flagellin (FliC) paralogs containing the N-terminal conserved flg22 motif were found in the genome of SA187. This Flg22 motif is the main pathogen-associated molecular pattern (PAMP) motif that induces PAMP-triggered immunity when plants sense bacteria, such as *P. aeruginosa* or *S. enterica* (García and Hirt, 2014; Bigeard et al., 2015) (**Figure 7B**). Since different host plants including Arabidopsis recognize SA187 as a beneficial bacterium and not a pathogen, it is clear that FliC-induced PAMP-triggered defense responses must be suppressed by SA187 through a yet unknown mechanism.

#### Plant Colonization

To mediate the adhesion and colonization of plant roots, a variety of plant-associated bacteria produce cellulose and other exopolysaccharides (Römling and Galperin, 2015). The genome of SA187 contains all genes necessary to synthesize cellulose (*bcsABCD*) (Supplementary Table S15). In some members of the *Enterobacteriaceae*, cellulose is usually co-expressed with curli fibers (Römling, 2007). In the genome of SA187 we found 2 operons coding for curli fibers: *csgGFED* (SA187PBcda_000003728-000003731) and *csgBAC* (SA187PBcda_000003733-000003735), which were found adjacent, separated by a hypothetical protein (SA187PBcda_000003732).

Colanic acid is another exopolysaccharide produced by many *Enterobacteriaceae* and critical for biofilm formation (Rättö et al., 2006). The genome of SA187 contains all genes necessary for the biosynthesis of colanic acid, organized in a gene cluster, together with the proteins required for its translocation to the bacteria cell surface: Wza, Wzb, and Wzc (SA187PBcda_000005052-000005070). The colanic acid transcriptional regulator McbR (SA187PBcda_000004274) was also identified in a separate genomic region. Additionally, we found several genes involved in the putative adhesion to roots, as well as the operon *srfABC* (SA187PBcda_000004281-000004283), which codes for virulence effectors homologs to those identified in *S. enterica*, which are believed to be involved in host colonization (Worley et al., 2000; Frye et al., 2006).

It has been reported that the endophytic bacterium *E. asburiae* JM22 is able to hydrolyze plant cell wall-bound cellulose to gain access to the plant cell (Hallmann et al., 1997). The genome of SA187, however, does not encode any endo-/exo- or hemi-cellulases, suggesting that SA187 could gain entry into the host plant through injuries, points of damaged tissue or natural openings, as described for *Enterobacter* sp. 638 (Taghavi et al., 2010). Similarly to this well characterized PGPB, we found that SA187 is able to degrade and utilize pectin, as a gene coding for pectate lyase (*pelB*, SA187PBcda_000002287), an enzyme involved in the cleavage of pectate into oligosaccharides, was identified next to an oligogalacturonate-specific porin (*kdgM*, SA187PBcda_000002288) (Taghavi et al., 2010). We also found an oligogalacturonide ABC transporter (*togMNAB*, SA187PBcda_000000314-000000317) involved in the translocation of this pectic oligosaccharide across the inner membrane and genes involved in the degradation of oligogalacturonide (*ogl*, SA187PBcda_000000332; *kduDI*, SA187PBcda_000000330-000000331; *kdgKA*, SA187PBcda_000001733-000001734), was well as the transcriptional regulator *kdgR*, SA187PBcda_000001735) and 2 copies of an additional galacturonate uptake transporter *exuT* (SA187PBcda_000000661, SA187PBcda_000003517), and its negative regulator *exuR* (SA187PBcda _000000662) (Valmeekam et al., 2001). This strategy has been also reported for others PGPB, such as *Bacillus amyloliquefaciens* subsp. *plantarum* B9601-Y2 (He et al., 2012). Alternatively, we found that SA187 may degrade galacturonate through a pathway involving the enzymes coded by *uxaA* (SA187PBcda_000000658), *uxaB* (SA187PBcda_000004197-000004198), and *uxaC* (SA187PBcda_000000659). Enzymatic complexes UxaABC and Uxa AB has been found to be normally used in *E. coli* to degrade galacturonate and glucuronate (Rothe et al., 2013).

As described previously, carotenoids play an important role in the survival in the rhizosphere and plant colonization of *Pantoea* sp. YR343 to *A. thaliana* and *Populus deltoids* (Bible et al., 2016). Interestingly, during our experiments to screen the effect of SA187 in the growth and development of Arabidopsis seedlings, we monitored the amount and viability of bacterial cells (CFU/μl) that were associated with plant roots, and observed the appearance of a multiphenotypic complex in showing yellow and white colonies (**Figure 6B**). The ratio between white colonies (SA187W) and those showing the original yellow phenotype (SA187Y) increased with longer periods of incubation with plants (data not shown). To eliminate the possibility of contamination, we performed analysis of the 16S rRNA gene sequence as well as genotyping of both white and yellow isolates by (GTG)_5_-rep-PCR fingerprinting. The results obtained from these analyses confirmed both morphologies corresponded to the same bacterial strain (**Figure 6C**). Based on these results, we can hypothesize that a modification in the metabolism of SA187 occurred upon colonization of the plant, leading to a decrease in the production of carotenoids. Although a clear role of carotenoids in the interaction between SA187 and Arabidopsis was suggested by the increase in gene expression of *crtB* upon association with the plant (**Figure 4A**), further investigations are needed to clarify a role of these carotenoids in the interaction of SA187 with its host–plant.

#### Defense against Oxidative Stress

Upon contact with bacteria, a major plant defense reaction is the production of reactive oxygen species (ROS), nitric oxide and phytoalexins (Hammond-Kosack and Jones, 1996; Zeidler et al., 2004). Therefore, during colonization, endophytes have to survive in a highly oxidative environment. Accordingly, in the genome of SA187 we found a wide variety of enzymes and regulators that help bacteria to cope with oxidative stress, including superoxide dismutase (*sod*, SA187PBcda_000001593), catalase (*katE*, SA187PBcda_000005240), Mn-containing catalase (*katN*, SA187PBcda_000000181), 4 peroxiredoxins (*ahpC*, SA187PBcda_000002814, SA187PBcda_000003138; *bcp*, SA187PBcda_000005379; *ahpF*, SA187PBcda_000003139), 2 osmotically inducible proteins (*osmC*, SA187PBcda_000000190, SA187PBcda_000002308), iron-dependent peroxidase (SA187PBcda_000003030), cloroperoxidase (SA187PBcda_000001174) and thiol peroxidase (SA187PBcda_000004379). Synteny analysis demonstrated that the gene SA187PBcda_000003918, annotated as hypothetical protein, was in reality a homolog of *katE*.

Additionally, we found a gene coding for the hydrogen peroxidase sensor OxyR (SA187PBcda_000001637), which activates the expression of genes such as glutathione reductase (*katG*, gor, SA187PBcda_000001159), *ahpC*, *ahpF*, a DNA-protection during starvation protein (*dpsA*, SA187PBcda_000003479), the transcriptional regulator of ferric uptake (*fur*, SA187PBcda_000003300) and glutaredoxin (*grxA*, SA187PBcda_000003553). We also found 3 glutathione *S*-transferases (GTS) (*gts*, SA187PBcda_000001274, SA187PBcda_000002004, SA187PBcda_000003538), a glutathione ABC transporter (*gsiABCD*, SA187PBcda_000003528-000003531), 3 glutathione peroxidases (*btuE*, SA187PBcda_000003945, SA187PBcda_000004145) and a γ-glutamyl transpeptidase (GGT) (SA187PBcda_000001086). The operon coding for an RND family multidrug efflux pump (*acrAB*, SA187PBcda_000002911-000002912) that is required for a successful colonization of the host plant (Taghavi et al., 2010; Burse et al., 2004), as well as its transcriptional regulator (*acrC*, SA187PBcda_000002913), were also encoded in the genome of SA187.

#### Production of Antimicrobial Compounds and Toxins

Many beneficial bacteria also produce a variety of antimicrobial compounds, thereby enhancing the plant resistance against pathogens. The genome of SA187 contain *phzF* (SA187PBcda_000004183) and *ubiC* (SA187PBcda_000001974), two enzymes involved in the biosynthesis of phenazine and 4-hydroxybenzoate, respectively, which are antibiotics against plant pathogenic bacteria (Duan et al., 2013; Gupta et al., 2014). Furthermore, we identified six genes coding for chitinase (gene.SA187PBcda_000000184, gene.SA187PBcda_000000547,gene.SA187PBcda_000000978, gene.SA187PBcda_000000980, gene.SA187PBcda_000003882, gene.SA187PBcda_000005622), a potent enzyme against insects and fungi (Hamid et al., 2013), which has been also identified in PGPB of the genera *Enterobacter*, *Klebsiella*, *Pantoea*, or *Serratia* (Dinesh et al., 2015; Rodrigues et al., 2016). SA187 can also synthetize proteins involved in resistance against several antimicrobial compounds: ß-lactam, vancomycin and cationic antimicrobial peptide (CAP) (Supplementary Table S16). We also found a number of toxin/antitoxin (T/A) systems (*symE/R*, *relE/B*, *hipA/B*, *cptA/B*, *chpBK/BI*, *vapB/C*, *fic/yhfG*, *hicA/B*) (Supplementary Table S17) and the toxin-coregulated proteins TcpA (gene.SA187PBcda_000000329) and TcpE (gene.SA187PBcda_000000321), what is consistent with the dualistic life-style of SA187 as free-living in the rhizosphere and associated with the host–plant (Pandey and Gerdes, 2005).

#### Plant Hormone Modulation and Promotion of Plant Growth

Many beneficial bacteria have PGPB activity that is mediated by a variety of mechanisms, including the production or inactivation of plant hormones, such as aminocyclopropane-1-carboxilate (ACC) deaminase. ACC deaminase is involved in the metabolism of the immediate precursor of ethylene in the ethylene biosynthesis, and one of the most well-known PGPB traits (Loper et al., 2012; Shen et al., 2013). ACC deaminase has been found predominantly in *Pseudomonas* and *Mesorhizobium* strains, but also reported in member of the genus *Enterobacter*: *E. cloacae* UW4, *E. cloacae* CAL2, and rhizospheric *E. cloacae* and *E. cancerogenus*, among others (Shah et al., 1998; Holguin and Glick, 2001; Glick, 2014). Interestingly, *acdS*, coding for ACC deaminase, was not found in the genome of SA187 (Supplementary Figure S6).

Acetoin and 2,3-butanediol are volatile organic compounds (VOCs) emitted by many PGPB to enhance plant growth (Ryu et al., 2003). The main pathway for the production of these VOCs by *Enterobacter* sp. 638, a PGPB strain closely related to SA187, is via the sequential action of enzymes coded by the operon *budABC* (Taghavi et al., 2010). The genome sequence of SA187 contains 3 paralogs of the dimeric enzyme coded by *budB* (SA187PBcda_000001378-000001379, SA187PBcda_000001698-000001699, SA187PBcda_000002514-000002515), acetolactate synthase, but no *budA* or *budC* are present. The lack of these genes makes SA187 capable to transform pyruvate into acetolactate, but no further transformation into acetoin or 2,3-butanediol is possible.

Another strategy that PGPB use to enhance plant growth is the synthesis of auxin indole-3-acetic acid (IAA) from tryptophan through indolepyruvate (Taghavi et al., 2009). We found that SA187 contains most of the genes coding for enzymes involved in this pathway, but it lacks the gene coding for indolepyruvate decarboxylase (*ipdC*). Instead, SA187 encodes the enzyme tryptophanase (*tnaA*, SA187PBcda_000000047), which was also found in biofilm forming *E. coli* (Hu et al., 2010), which can transform tryptophan into indole, but cannot produce salicylic acid (SA). There is nevertheless the possibility that IAA could be produced from indole, although the mechanism of this reaction is not yet understood. We also found a gene coding for nitrilase (SA187PBcda_000002715), which could be a possible alternative tryptophan-independent pathway for the biosynthesis of IAA from indole-3-acetonitrile (Bhattacharyya et al., 2017). Alternatively, SA187 could supply the plant with tryptophan itself, which is the source for the *de novo* synthesis of IAA in plants, through the intermediate indole-3-pyruvate (Zhao, 2012).

Additionally, we found that the genome of SA187 also contains genes coding for arginine decarboxylase (SpeA), agmatinase (SpeB) and spermidine synthase (SpeE) (*speABE*, SA187PBcda_000002462-000002464). These enzymes allow the transformation of amino acids into PGP substances, the polyamines putrescine, spermine, and spermidine, respectively, which contribute to bacterial fitness, and have been reported in PGPB strains such as *B. subtilis* OKB105 or *Klebsiella* sp. D5A (Xie et al., 2014; Liu et al., 2016).

Although SA187 lacks the former common PGP mechanisms, its beneficial effect in promoting the growth of plants under stress has been suggested to be due to the production of ethylene. Several mechanisms have been described for the production of ethylene in microbes, as ethylene-forming enzyme (EFE) or spontaneous oxidation of 2-keto-4-methylthiobutyric acid (KMBA), an intermediate of the methionine salvage pathway (MSP) (Eckert et al., 2014). In the genome of SA187, we identified all genes involved in the MSP, suggesting that SA187 has the potential to produce ethylene in this manner (Supplementary Figure S6).

#### SA187 Central Metabolism

The genome of SA187 contains genes involved in the central carbon metabolism, including glycolysis (Embden–Meyerhof and Entner–Doudoroff pathways), pyruvate oxidation, tricarboxylic acid cycle, pentose phosphate pathway and glyoxylate cycle (Supplementary Table S18). The presence of these metabolic pathways should provide SA187 with the capacity to metabolize sugars and other carbon sources present in the plant root exudates. We also found that SA187 can utilize lactose, a differentiating characteristic of the genera *Escherichia, Enterobacter, Citrobacter, Klebsiella*, and *Serratia* (Guentzel, 1996), and also fructose, mannose and malonate (*mdcABCDEFGH*, SA187PBcda_000000997-000001004; and the transcriptional regulator *mdcR*, SA187PBcda_000000996). The capacity of SA187 to metabolize these sugars is consistent with its uptake through ABC transporters or PTS, as described before, and with the capability of SA187 to grow in ½ MS+1% fructose and ½ MS+0.1–1% sucrose (Supplementary Figure S5B). The role of sucrose as carbon source utilized by the bacteria in association with the plant is also supported by the observed increase in gene expression of the sucrose transporter *scrA* (**Figure 4A**).

## Conclusion

The results of our taxonomic analysis supports the notion that SA187 represents a novel taxon closely related to the genus *Kosakonia*. Although this suggests the classification of SA187 as a new genus, further investigation is needed to fully characterize its taxonomic position. Additionally, in the genome of SA187, we successfully identified a number of genes conferring the bacterium the characteristics of a dualistic life style, allowing SA187 to survive in the soil under harsh conditions, as well as to colonize, internalize and provide growth promotion to plants through diverse metabolic strategies. However, the mechanism of interaction between SA187 and its host-plant is not yet fully understood. The combination of the present genomic data with comparative studies on gene expression and metabolite production in SA187, alone or in association with plants, will deepen our understanding which specific genes and pathways are induced during the beneficial interaction. Once important genes will be identified, the further phenotypic screening of mutant strains will reveal their role in the plant-bacterial interaction and plant growth promotion. The knowledge obtained can be further translated into comprehensive and more sophisticated strategies to establish sustainable agricultural practices in marginal and arid lands by using endophytic bacteria as biofertilizers to improve crop production.

## Author Contributions

MS, FL, and HH conceived the overall study. AdZ established the plant screening assay that led to the selection of SA187. FL extracted and purified SA187 genomic DNA. IA performed gene prediction and annotation of complete genome data. CA-B performed taxonomical analysis based on 16S and MLSA. FL and IA performed whole-genome phylogenetic analysis. AE performed the biochemical characterization of SA187 and growth curves. AB realized the plant growth promotion screening. HA extracted RNA from dual samples CA-B extracted RNA from bacterial samples, performed qPCR and analyzed the genomic data. CA-B and MS wrote the manuscript. HH, VB, and MS coordinated the experiments and the writing of the manuscript.

## Conflict of Interest Statement

The authors declare that the research was conducted in the absence of any commercial or financial relationships that could be construed as a potential conflict of interest.

## Footnotes

1 http://www.darwin21.net

2 http://genskew.csb.univie.ac.at

3 https://www.ncbi.nlm.nih.gov/nucleotide/
