# Supplementary figures and images for: Complete Genome Sequence Analysis of Enterobacter sp. SA187, a Plant Multi-Stress Tolerance Promoting Endophytic Bacterium
Source: Front Microbiol. 2017 Oct 20;8:2023. doi: 10.3389/fmicb.2017.02023 (PMC5664417; doi:10.3389/fmicb.2017.02023)

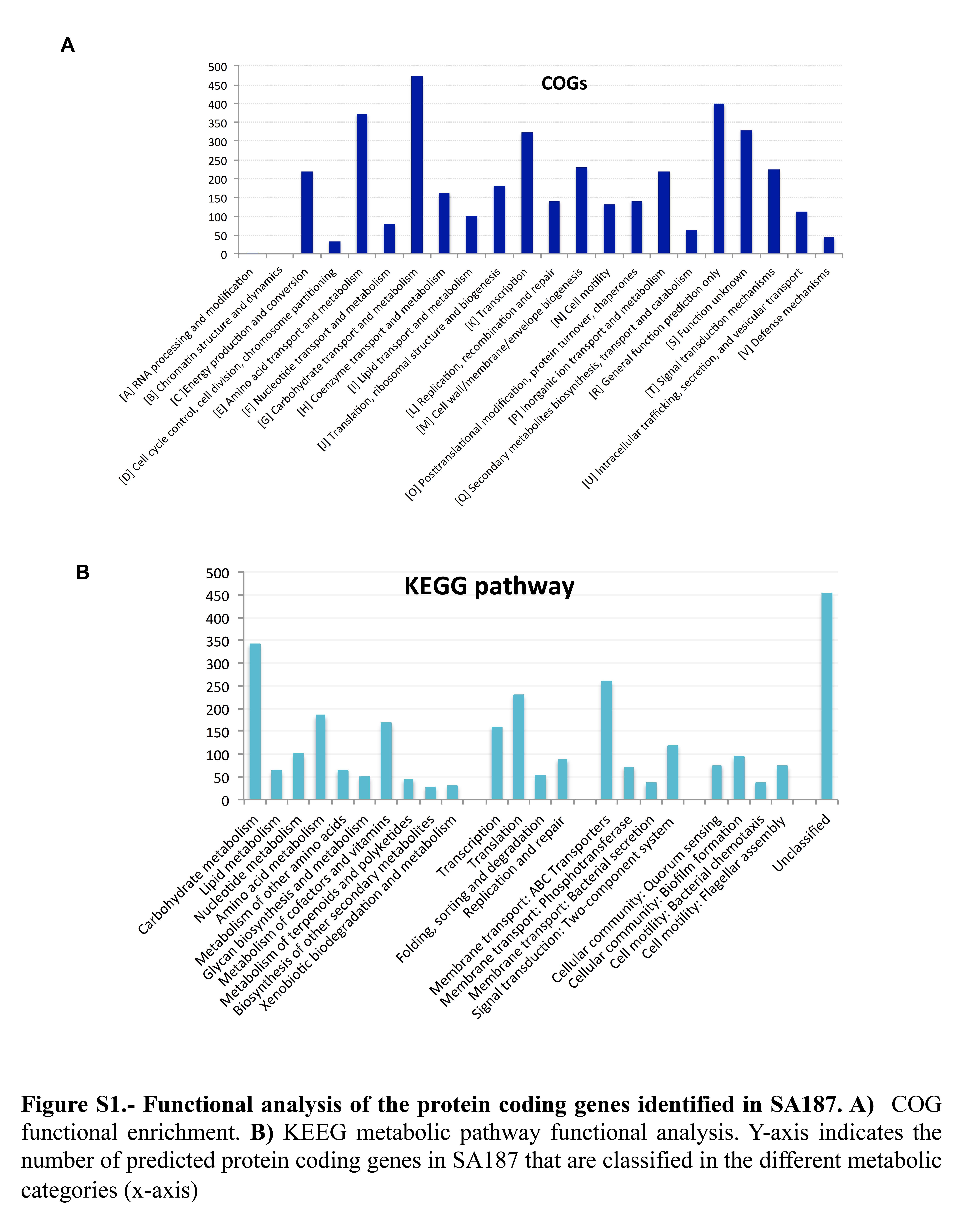

Supplement: Supplementary file 2 [file Image_1.TIFF]

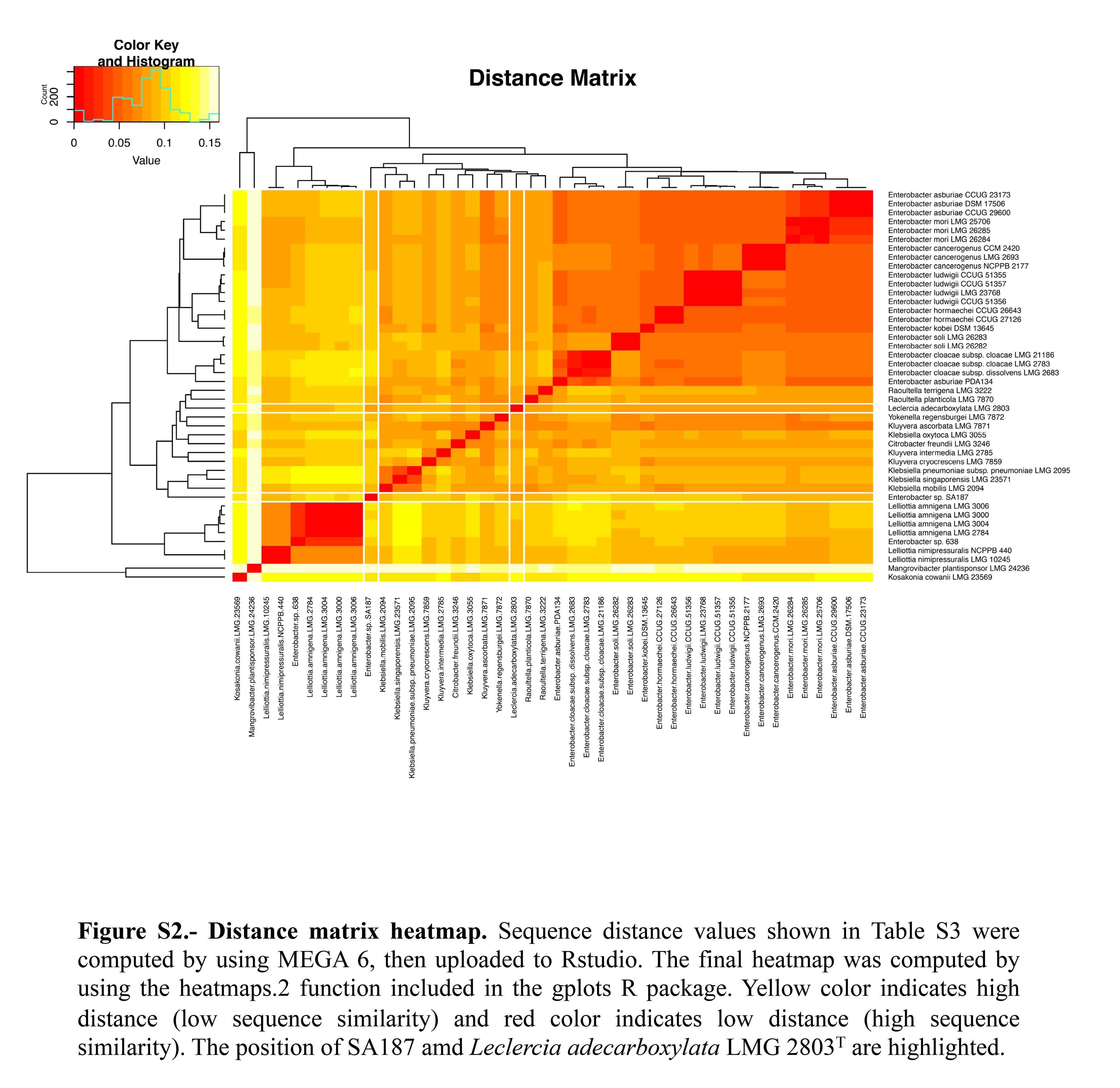

Supplement: Supplementary file 3 [file Image_2.TIFF]

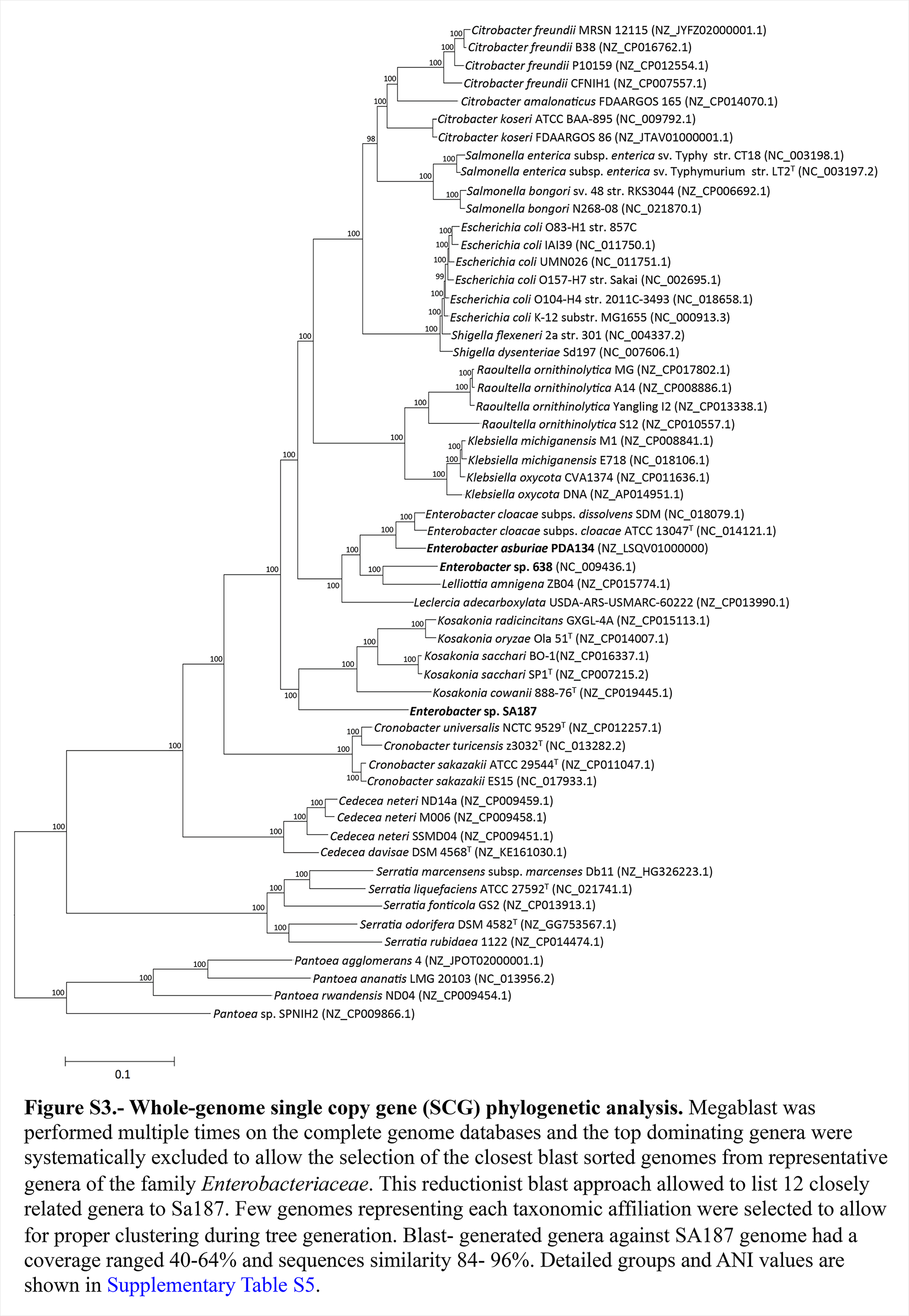

Supplement: Supplementary file 4 [file Image_3.TIF]

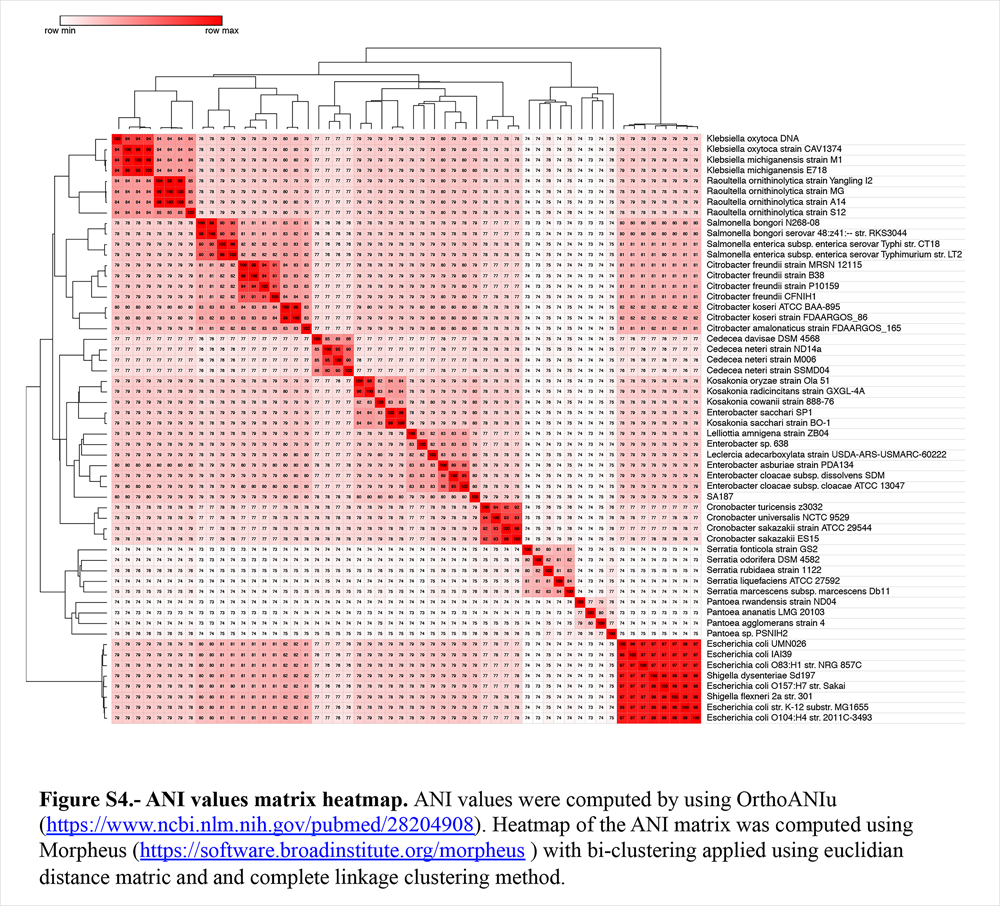

Supplement: Supplementary file 5 [file Image_4.TIF]

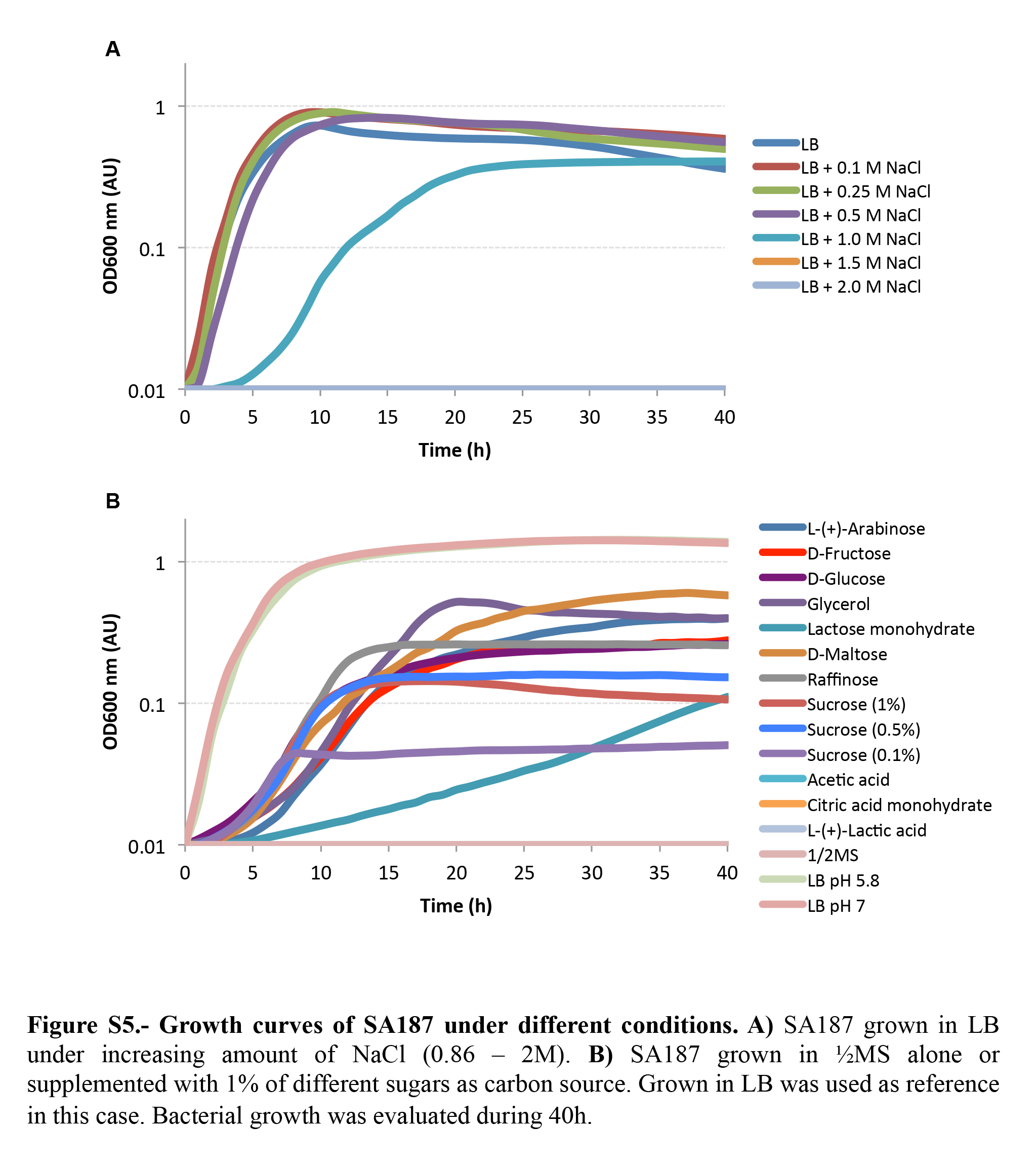

Supplement: Supplementary file 6 [file Image_5.TIF]

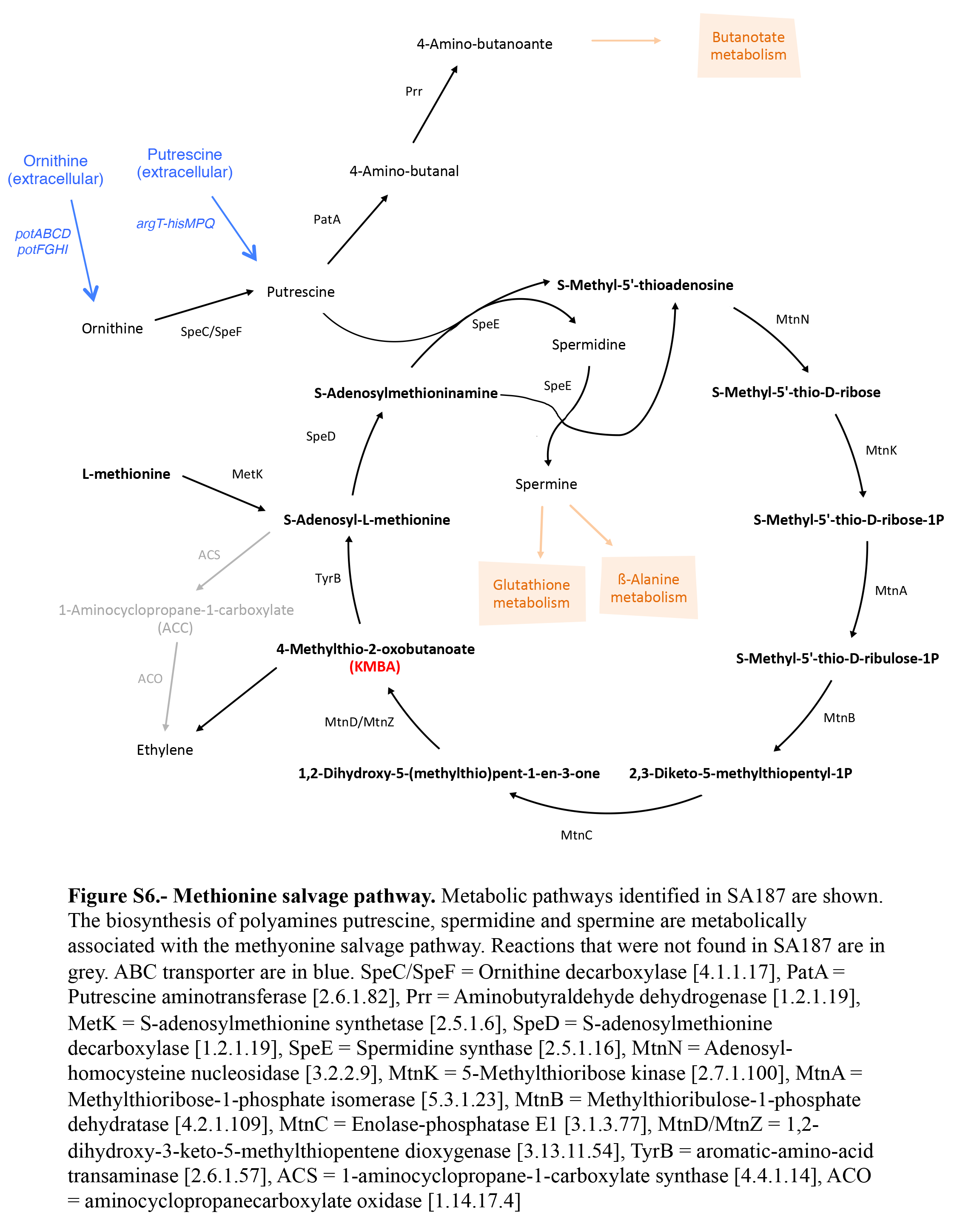

Supplement: Supplementary file 7 [file Image_6.TIF]
